# Why Circulating Interleukin‐6 Is Not Yet a Standalone Clinically Actionable Biomarker in HFrEF: A Context‐of‐Use Review

**DOI:** 10.1111/eci.70261

**Published:** 2026-10-17

**Authors:** Yongxin Ye, Martina Colombo, Jennifer Meessen, Benoit Tyl, Deborah Novelli, Negin Hajizadeh, Hans‐Peter Brunner‐La Rocca, Adriaan Voors, Sara Klas, Stephan Schwers, Bjarke Follin, Roberto Latini

**Affiliations:** ^1^ Novo Nordisk A/S Søborg Denmark; ^2^ Department of Acute Brain and Cardiovascular Injury Istituto di Ricerche Farmacologiche Mario Negri IRCCS Milan Italy; ^3^ Bayer HealthCare SAS La Garenne Colombes France; ^4^ Bayer Espana AG Madrid Spain; ^5^ Cardiovascular Research Institute Maastricht (CARIM) Maastricht University Maastricht the Netherlands; ^6^ Department of Cardiology Maastricht University, Medical Center MUMC+ Maastricht the Netherlands; ^7^ Department of Cardiology, University Medical Center Groningen University of Groningen Groningen the Netherlands; ^8^ Evotec International, GmbH Hamburg Germany; ^9^ Bayer AG Wuppertal Germany

**Keywords:** heart failure with reduced ejection fraction, interleukin‐6, pharmacodynamic, predictive, prognostic, surrogate

## Abstract

**Background:**

Interleukin‐6 (IL‐6) is frequently measured in heart failure with reduced ejection fraction (HFrEF), but its clinical utility remains unclear.

**Objectives:**

To assess IL‐6 as a biomarker in HFrEF, with emphasis on the effect of treatments (pharmacodynamics) and translation readiness.

**Methods:**

MEDLINE and EMBASE were searched through June 2026. Using AI‐assisted full‐text screening with manual adjudication, eligible studies were classified using BEST classification as prognostic, predictive and/or pharmacodynamic biomarkers.

**Results:**

Eighty studies met inclusion criteria (59 pharmacodynamic, 21 prognostic, 3 predictive; categories overlapping). Across prognostic studies, higher IL‐6 consistently tracked disease severity and cardiovascular mortality risk, but associations with hospitalization and composite endpoints weakened after adjustment for natriuretic peptides, limiting incremental clinical value. Pharmacodynamic studies showed that several interventions reduce circulating IL‐6—most consistently statins and levosimendan—while findings were heterogeneous for exercise, cardiac device, and sodium–glucose co‐transporter‐2 inhibitors. However, IL‐6 changes did not consistently parallel clinical benefit, arguing against its use as a surrogate measure for outcome improvement. No study supported IL‐6 as a predictive biomarker. Assay heterogeneity was substantial, with limited use of in vitro diagnosis‐grade assays, representing a major barrier to qualification.

**Conclusions:**

In HFrEF, IL‐6 reflects inflammatory burden and may serve as pharmacodynamic readout in mechanistic studies, but it is not supported for treatment selection and adds limited prognostic information beyond natriuretic peptides. Progress towards translational utility requires standardized measurements and, potentially, biomarkers reflecting the IL‐6 axis beyond IL‐6 alone.

## Highlights

- IL‐6 tracks inflammatory burden and mortality risk in HFrEF, but its incremental prognostic value beyond established HF biomarkers is limited.
- IL‐6 changes may reflect system response depending on intervention mechanism.
- Current evidence does not support IL‐6 as a biomarker predicting response to heart failure treatment.

## Graphical abstract

Clinical and translational readiness of IL‐6 measurement in HFrEF.

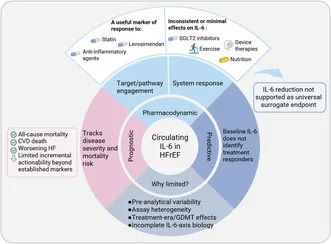

**Abbreviations**
HFpEF: heart failure with preserved ejection fraction
HFrEF: heart failure with reduced ejection fraction
IL‐6: interleukin‐6

## 1 Introduction

Over the past 30 years, the number of circulating biomarkers proposed to support clinicians in the diagnosis, prognosis and treatment of cardiovascular diseases has increased markedly, yet only a few have entered clinical practice. This is especially true for heart failure (HF) where only N‐terminal pro B‐type natriuretic peptide (NT‐proBNP) has been included in the prognosis and management [1]. While natriuretic peptides are excellent biomarkers for prognosis and management, they do not capture the full complexity of HF pathophysiology.

Inflammation is a key contributor to the pathogenesis and progression of HF. Interleukin‐6 (IL‐6), a pleiotropic cytokine and the main regulator of hepatic C‐Reactive Protein (CRP), has gained attention due to its established role in cardiovascular diseases. IL‐6 is synthetized during the early stages of inflammation and drives downstream proinflammatory responses. Sub‐analysis of the CANTOS trial and many experimental studies have identified IL‐6 as a major contributor to atherothrombotic disease [2]. In HF, IL‐6 may influence disease progression through immune activation, myocardial remodelling, and systemic inflammation [3]. Accordingly, IL‐6 has been proposed as both a biomarker and a potential therapeutic target. Repeated IL‐6 measurements have been proposed to guide HF treatment [4], and higher IL‐6 levels have been associated with worse outcomes in large cohorts such as BIOSTAT‐CHF [5]. Genetic studies further support a causal role of the IL‐6 pathway in cardiovascular risk, providing a rationale for therapeutic inhibition [6]. Consistent with this, monoclonal antibodies targeting the IL‐6 ligand, such as ziltivekimab [7] and clazakizumab [8], are currently under clinical investigation in populations with cardiovascular and renal disease.

Although IL‐6 has been measured in numerous HFrEF studies, its practical utility—particularly as a biomarker of treatment response—remains unclear. The extensive evidence base spanning both effective and ineffective interventions in HFrEF provides a unique opportunity to evaluate IL‐6 not only as a prognostic biomarker, but also as a pharmacodynamic (treatment‐response) and predictive biomarker.

This review addresses a specific question: whether circulating IL‐6 measurement in HFrEF meets biomarker standards for defined contexts of use. We therefore reviewed the relevance of IL‐6 as a biomarker in HFrEF using the FDA‐NIH BEST (Biomarkers, EndpointS, and other Tools) framework [9], focusing on its performance across three contexts of use: prognostic (risk stratification), pharmacodynamic (treatment‐induced changes), and predictive (identification of treatment responders). By mapping these complementary applications, we aimed to clarify whether IL‐6 provides actionable information for clinical decision‐making or drug development, and to explore whether treatment‐related IL‐6 changes are sufficiently concordant with clinical benefit to support it as a surrogate biomarker in HFrEF.

## 2 Methods

### 2.1 Search Strategy and Selection Criteria

This review was conducted in accordance with the Preferred Reporting Items for Systematic Reviews and Meta‐Analyses (PRISMA) 2020 guidelines [10]. Eligibility criteria were defined using the PICO (Population, Intervention, Comparison, Outcome) framework to establish inclusion and exclusion criteria (Table S1). The population of interest consisted of patients with heart failure with reduced ejection fraction (HFrEF), defined as left ventricular ejection fraction (LVEF) < 50% according to the 2026 ESC Guidelines for the management of heart failure [1], or patients receiving advanced therapies such as left ventricular assist devices (LVADs) or cardiac resynchronization therapy (CRT), or described as having systolic heart failure. Studies were also included if they reported subgroup analyses specifically focusing on HFrEF. The primary focus was on IL‐6 as a biomarker of interest. Studies including biomarker analyses in the specified population were considered eligible. Exclusion criteria included studies focusing on heart failure with preserved ejection fraction (HFpEF; LVEF ≥ 50%), new‐onset heart failure, heart transplantation, and congenital heart disease. A comprehensive literature search was performed in MEDLINE and EMBASE from database inception to June 2026 using predefined search strings (see Supporting Information). No initial restrictions on study design were applied. Relevant narrative reviews, systematic reviews and meta‐analyses were used as supplementary sources to identify studies not captured by the main search strategy. The references listed in these reviews were manually screened, and those meeting the inclusion criteria were added to the pool of publications.

### 2.2 Study Selection Process

An artificial intelligence (AI)‐based tool leveraging a large language model (LLM) was developed by Capsico Health (Palo Alto, CA, United States) to automate and expedite the screening of publications identified. Unlike the traditional title and abstract screening, the AI assessed inclusion and exclusion criteria directly from full texts. The AI tool was trained and validated using a set of publications previously assessed by human reviewers, and its performance was benchmarked against human assessment. Settings were optimized to minimize false negatives, accepting more false positives that could subsequently be reviewed manually. Once validated, the tool was used to screen all full texts retrieved. A detailed description of the development, training and performance of the AI tool has been published elsewhere [11].

### 2.3 Data Extraction

Publications deemed eligible by the AI‐based tool according to our criteria were subsequently manually classified into three predefined biomarkers categories: prognostic, pharmacodynamic and predictive (Table S2). A single study could address multiple biomarker roles and thus contribute to more than one category. Besides, the studies were also assessed as a surrogate biomarker by comparing treatment‐induced IL‐6 changes with available clinical outcome evidence. Some studies, although meeting the inclusion and exclusion criteria, did not fit into any of the three categories of interest and were therefore excluded. This classification was performed by a group of reviewers and in case of uncertainty, consensus was reached through group discussion. As part of the classification phase the following data were manually extracted from the selected articles: (i) general study information: author, year, study design (prospective or retrospective); (ii) patient characteristics: sample size, age; (iii) biomarker measurement: time point of measurement, assay; (iv) follow‐up duration (v) outcome measures (i.e., hazard ratio, c statistic, etc.). Risk of bias for prognostic studies was assessed using the QUIPS tool [12], while no validated instruments were available for pharmacodynamic or predictive studies.

## 3 Results

### 3.1 Study Selection and Assay Characteristics

After de‐duplication and removal of inaccessible records, 415 publications on IL‐6 were identified, along with 54 review articles. Of the 415 studies, 24 were manually screened, resulting in the inclusion of 1 study. Additionally, 10 publications were not available in PDF format and were therefore screened manually; this led to the inclusion of 2 further studies. Two additional relevant studies were identified through manual checking of literature. Overall, the AI tool successfully screened 381 publications, of which 77 were flagged as candidates for inclusion.

Of the 54 literature reviews, 18 were included after manual assessment. Screening these reviews and their reference lists yielded 37 additional studies, leading to 119 eligible publications overall. After applying inclusion/exclusion criteria (Table S1) and category assignment (Table S2), 39 were excluded because they did not fit into any of the three categories or did not meet eligibility criteria. This resulted in 80 single studies included and classified as pharmacodynamic (*n* = 59), predictive (*n* = 3), and prognostic (*n* = 21) with some overlap across categories. The selection process is shown in Figure 1.

**FIGURE 1:**
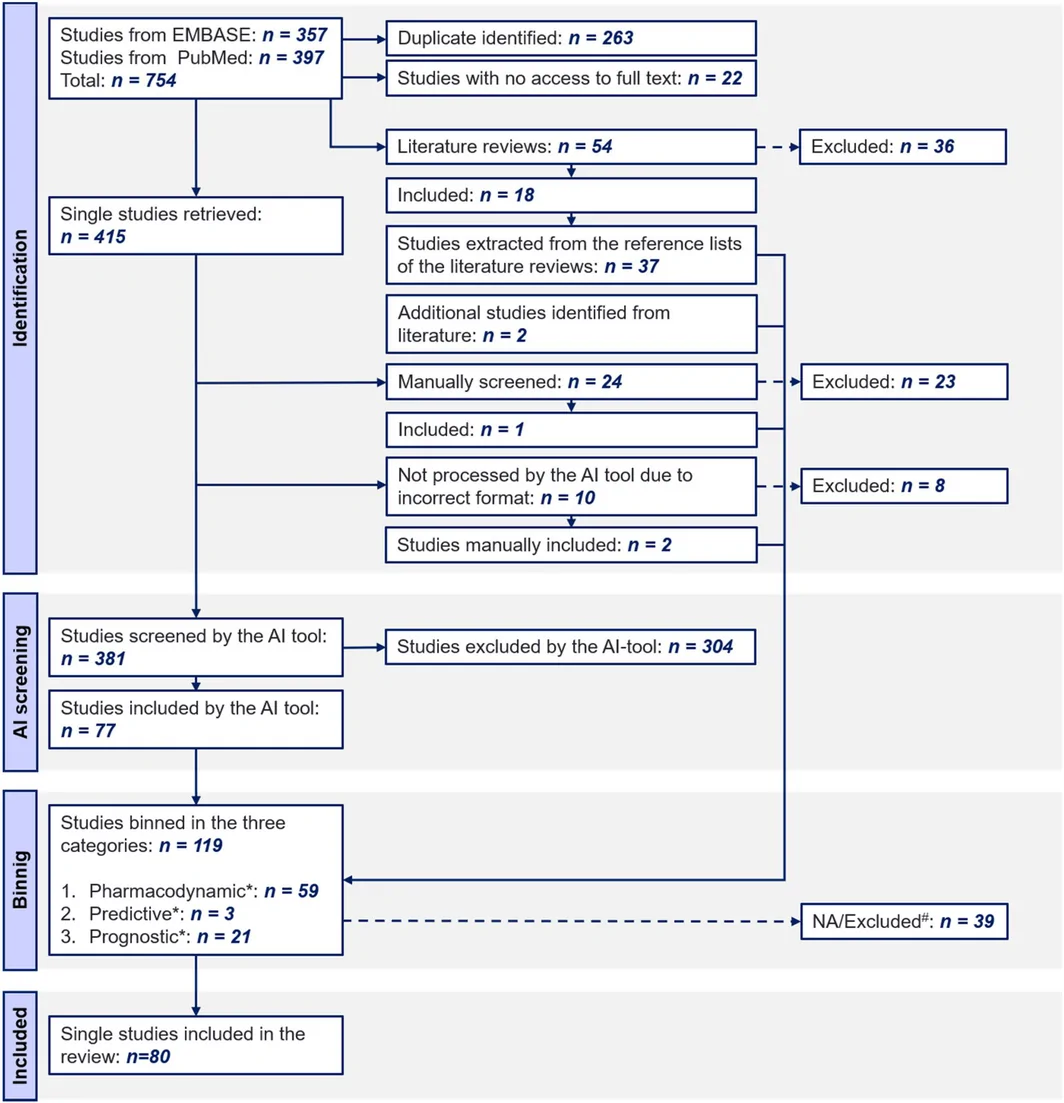
PRISMA flow diagram of the study selection process. *Some publications fit more than one category. ^#^NA/Excluded: Publications excluded for not meeting inclusion/exclusion criteria or category requirement.

Assays used across studies are summarized in Table S3 together with pre‐analytical procedures. Despite standardized IL‐6 assays being available since 2000, many studies employed research‐use‐only reagents, while only a few used In Vitro Diagnostics assays. Considerable variability was observed in pre‐analytical procedures, including specimen matrix, anticoagulant use, sample handling and storage conditions.

### 3.2 IL‐6 as a Prognostic Marker of Clinical Outcome in HF

Twenty‐one publications investigated IL‐6 as a prognostic marker (Table S4) including five randomized controlled trials (RCTs) [13, 14, 15, 16, 17] (total *n* = 9889) and 16 prospective cohort studies (total *n* = 4179). The largest cohort was BIOSTAT‐CHF (*n* = 2329) [5, 18, 19]. Mean follow‐up was 21.9 months [15, 20, 21, 22, 23]. The main outcomes were all‐cause mortality, cardiovascular (CV) death, hospitalization for worsening HF, and different HF composites (Table 1). Details of the risk of bias assessment using QUIPS are reported in Table S5.

**TABLE 1:** Summary of literature on IL‐6 as prognostic biomarker in patients with HFrEF.

References	*n*	% female	Additional characteristics when specified	FU	Study design	Univariate analysis	Univariate results	Multivariable analysis	Multivariable results	Adjustment
*All cause death*										
Torre‐Amione et al. (1996)	63	11%	Subset of patients enrolled in the neurhormonal subsidies in SOLVD	48 months	RCT	—	—	Not significant	*χ* ^2^ = 0.6 *p* = 0 = 0.72	TNF‐alpha, PNE, ANP, angiotensin‐converting enzyime inhibitor
Tsutamoto et al. (1998)	100	25%	Patients with congestive HF who were without renal failure, infection, malignancy or collagen disease and who underwent cardiac catheterization for clinical indications	28 months, range 3–54	Cohort, prospective	Significant	*χ* ^2^ = 18.8 *p* < 0.0001	Significant	*χ* ^2^ = 19.01 *p* < 0.0001	NE, ANP, NYHA class, LVEF, age, sex, aetiology (DCM, non‐DCM)
Maeda et al. (2000)	102	36%	Patients with severe congestive HF	807 days	Cohort, prospective	Significant	*χ* ^2^ = 4.08 *p* = 0.0435	Not significant	*χ* ^2^ = 2.40 *p* = 0.12	Age, sex, IHD, NYHA, LVEF, ANP, BNP NE, ET‐1, TNF‐alpha
Rauchhaus et al. (2000)	152	Not reported	Patients with chronic HF	24 months	Cohort, prospective	Significant	Continuous RR = 2.287 (1.285–4.068) *p* = 0.005 Top Quartile vs. Lower 3 Quartiles cutoff value (pg/mL) = 29.71 RR = 2.115 (1.113–3.965) *p* = 0.02	Not significant	NR	sTNF‐R2, TNF‐alpha, sCD14
Deswal et al. (2001)	1169	Not reported	Patients with advanced HF	55 weeks	RCT	Significant	NR	Not significant	NR	Age, sex, NYHA class, sodium, TNF, sTNFR1, sTNRF2, sIL‐6R
Marcucci et al. (2006)	214	21%	Patients with HF at hospital admission for a de novo diagnosis or for a decompensated chronic HF	8.5 months mean	Cohort, prospective	—	—	Significant	HR = 8.9 CI = (1.4–58.1) *p* = 0.02	Age, sex, traditional cardiovascular risk factors, systolic left ventricular function, renal failure, NYHA class, haemoglobin, serum sodium
Tsutamoto et al. (2007)	125	24%	HF patients due to dilated cardiomyopathy, ischaemic heart disease or hypertensive heart disease	Mean 2.9 year	Cohort, prospective	Significant	*χ* ^2^ = 27.68 *p* < 0.0001	Significant	*χ* ^2^ = 12.07 *p* = 0.0005	NYHA class, age, ischaemic aetiology, LVEF, noreprinephrine, log BNP, log TNF‐alpha, CT‐1, soluble gp130
Boffa et al. (2009)	79	15%	Patients admitted due to worsening of chronic HF	17.2 ± 8.4 months	Cohort, prospective	Not significant	NR	Not significant	HR = 0.99 CI = (0.82–1.22) *p* = 0.95	BNP, NYHA class, creatinine, LVEF
Boxer et al. (2011)	60	28%	Patients with HF	48 months	Cohort, prospective	Significant	1.06 (1.00–1.11) *p* = 0.035	Not significant	HR = 0.98 CI = (0.84–1.14) *p* = 0.79	Age, hsCRP, frailty score
Askevold et al. (2013)	1452	23%	Patients from CORONA trial, aged ≥ 60 years with chronic HF attributed to ischemic heart disease	955 days mean	RCT	Significant	1.44 (1.32–1.58) *p* < 0.001	Not significant	HR = 1.11 CI = (0.95–1.28) *p* = 0.185	LVEF, NYHA functional class, age, body mass index, diabetes mellitus, sex, intermittent claudication, heart rate, ApoB/Apo A‐1 ratio, estimated glomerular filtration rate, NT‐proBNP and hsCRP
Bielecka‐Dabrowa et al. (2013)	68	21%	Patients with dilated cardiomyopathy	60 months	Cohort, prospective	Not significant	NR	Not significant	NR	—
Mommersteeg et al. (2016)	104	28%	HF patients with depressive symptoms	6.1 years mean	Cohort, prospective	—	—	Significant	HR = 1.75 CI = (1.18–2.0) *p* = 0.006	Depressive symptoms, NGAL, TNF‐alpha, sTNFr1, sTNFr2, hsCRP, leukocytes, L‐arginine, ADMA, SDMA, Isoprostane, L‐arginine/ADMA
Markousis‐Mavrogenis et al. (2019)	2329	26%	Post doc analysis of the BIOSTAT‐CHF index cohort	21 months	Cohort, prospective	Significant	1.31 (1.25–1.38) *p* < 0.001	Significant	HR = 1.22 CI = (1.16–1.29) *p* < 0.001	Age, sex, BMI, eGFR, co‐morbidities, HF medications
Docherty et al. (2025)	2940	22%	Patients with chronic HF	12 months	RCT	Significant	Baseline IL‐6 tertiles T1: ≤ 4.72 HR = 1 (ref) T2: 4.73–7.89 HR = 1.38 (1.01–1.88) *p* = 0.044 T3: ≥ 7.90 HR = 2.91 (2.20–3.84) *p* < 0.001 IL‐6 per 1 U increase (log) 1.73 (1.55–1.93) *p* < 0.001	Significant	Baseline IL‐6 tertiles T1: ≤ 4.72 HR = 1 (ref) T2: 4.73–7.89 HR = 1.16 CI = (0.85–1.59) *p* = 0.36 T3: ≥ 7.90 HR = 1.78 CI = (1.32–2.40) *p* < 0.001 IL‐6 per 1 U increase (log) HR = 1.37 CI = (1.20–1.58) *p* < 0.001	Age, sex, systolic blood pressure, heart rate, ejection fraction, previous HF hospitalization, diabetes status, atrial fibrillation, ischemic aetiology, eGFR, randomized treatment (dapagliflozin), log hs‐TnT, and log NT‐proBNP
*CV death*										
Askevold et al. (2013)	1452	23%	Patients from CORONA trial, aged ≥ 60 years with chronic HF attributed to ischemic heart disease	955 days mean	RCT	Significant	1.42 (1.28–1.57) *p* < 0.001	Not significant	HR = 1.05 CI = (0.89–1.24) *p* = 0.569	LVEF, NYHA functional class, age, body mass index, diabetes mellitus, sex, intermittent laudication, heart rate, ApoB/Apo A‐1 ratio, estimated glomerular filtration rate, NT‐proBNP and hsCRP
Markousis‐Mavrogenis et al. (2019)	2329	26%	Post doc analysis of the BIOSTAT‐CHF index cohort	21 months	Cohort, prospective	Significant	1.26 (1.19–1.34) *p* < 0.001	Significant	HR = 1.16 CI = (1.09–1.24) *p* < 0.001	Age, sex, BMI, eGFR, co‐morbidities, HF medications
Defilippi et al. (2023)	5050	24%	Post doc analysis of the VICTORIA trial	10.8 months mean	RCT	Significant	1.54 (1.45–1.62) *p* < 0.001	Significant	HR = 1.12 CI = (1.04–1.21) *p* = 0.005	ACE‐I/ARB, Albumin, Beta blocker, Bilirubin, Calcium, Chloride, Duration of primary HF diagnosis, GGT, Haemoglobin, History of anaemia, History of MI, History of PAD, History of PCI, ICD, Index event, NT‐proBNP at randomization, NYHA at baseline, Platelet, Heart rate, QTcF, Randomized while hospitalized, Systolic blood pressure, Urate
Docherty et al. (2025)	2940	22%	Patients with chronic HF	12 months	RCT	Significant	Baseline IL‐6 tertiles T1: ≤ 4.72 HR = 1 (ref) T2: 4.73–7.89 HR = 1.46 (1.04–2.05) *p* = 0.029 T3: ≥ 7.90 HR = 2.88 (2.12–3.91) *p* < 0.001 IL‐6 per 1 U increase (log) 1.69 (1.50–1.91) *p* < 0.001	Significant	Baseline IL‐6 tertiles T1: ≤ 4.72 HR = 1 (ref) T2: 4.73–7.89 HR = 1.20 CI = (0.85–1.69) *p* = 0.31 T3: ≥ 7.90 HR = 1.67 CI = (1.20–2.33) *p* = 0.002 IL‐6 per 1 U increase (log) HR = 1.29 CI = (1.11–1.51) *p* < 0.001	Age, sex, systolic blood pressure, heart rate, ejection fraction, previous HF hospitalization, diabetes status, atrial fibrillation, ischemic aetiology, eGFR, randomized treatment (dapagliflozin), log hs‐TnT, and log NT‐proBNP
*HF composites*										
Roig et al. (1998)	40	NA	Patients severe HF stabilized with medical therapy	18 ± 5 months	Cohort, prospective	—	—	Significant	OR = 1.04 CI = (1.07–1.01) *p* = 0.002	ANP, NYHA class
Maeda et al. (2000)	102	36%	Patients with severe congestive HF	807 days	Cohort, prospective	Not significant	*χ* ^2^ = 0.32 NS	Not significant	*χ* ^2^ = 0.11 *p* = 0.74	Age, sex, IHD, NYHA, LVEF, ANP, BNP, NE, ET‐1, TNF‐alpha
Orús et al. (2000)	87	NA	Patients with congestive HF	14 ± 9 months	Cohort, prospective	Significant	1.03 (0.6–1.58) *p* < 0.001	Significant	HR = 3.56 CI = (0.37–2.16) *p* = 0.01	—
Gwechenberger et al. (2004)	100	11%	Patients with congestive HF	Mean 378 days	Cohort, prospective	Significant	*χ* ^2^ = 19.92 *p* = 0.0001	Significant	*χ* ^2^ = 19.92 *p* = 0.0001	log BNP, log N‐ANP, log N‐BNP, norepinephrine, TNF‐alpha, LVEF, age, NYHA class
Marcucci et al. (2006)	214	21%	Patients with HF at hospital admission for a de novo diagnosis or for a decompensated chronic HF	8,5 months mean	Cohort, prospective	Not significant	NR	Not significant	NR	Age, sex, traditional cardiovascular risk factors, systolic left ventricular function, renal failure, NYHA class, haemoglobin, serum sodium
Boffa et al. (2009)	79	15%	Patients admitted due to worsening of chronic HF	17.2 ± 8.4 months	Cohort, prospective	Significant	NR	Not significant	HR =1.04 CI = (0.91–1.19) *p* = 0.56 continuous	BNP, NYHA class, creatinine, LVEF
Parissis, Farmakis, et al. (2009)	137	14%	Chronic HF patients with depressive symptoms	12 months/250 days	Cohort, prospective	Significant	NR	Not significant	NR	Age, sex, NYHA class, exercise capacity 6MWT, LVEF, depressive symptoms, BNP, IL‐10, TNF‐alpha
Parissis, Nikolaou, et al. (2009)	300	18%	Chronic HF patients	12 months	Cohort, prospective	Significant	1.837 (1.132–2.982) *p* = 0.014 Cutoff 5.8 pg/mL	Not significant	HR = 1.153 CI = (0.517–2.570) *p* = 0.729 cutoff 5.8 pg/mL	6MWT, BNP, NYHA class, IL‐10, TNF‐alpha
Gombos et al. (2012)	182	27%	Chronic HF patients	14.9 ± 6.8 months	Cohort, prospective	Not significant	NR	Not significant	NR	NT‐proBNP, BMI, diastolic blood pressure, haemoglobin, creatinine, sex, age
Askevold et al. (2013)	1452	23%	Patients from CORONA trial, aged ≥ 60 years with chronic HF attributed to ischemic heart disease	955 days mean	RCT	Significant	1.35 (1.23–1.49) *p* < 0.001	Not significant	HR = 1.02 CI = (0.88–1.19) *p* = 0.758	LVEF, NYHA functional class, age, body mass index, diabetes mellitus, sex, intermittent claudication, heart rate, ApoB/Apo A‐1 ratio, estimated glomerular filtration rate, NT‐proBNP and hsCRP
Markousis‐Mavrogenis et al. (2019)	2329	26%	Post doc analysis of the BIOSTAT‐CHF index cohort	21 months	Cohort, prospective	Significant	1.25 (1.20–1.30) *p* < 0.001	Significant	HR = 1.16 CI = (1.11–1.21) *p* < 0.001	Age, sex, BMI, eGFR, co‐morbidities, HF medications
Defilippi et al. (2022)	5050	24%	Post doc analysis of the VICTORIA trial	10.8 months mean	RCT	Significant	1.39 (1.33–1.45) *p* < 0.001	Significant	HR = 1.07 CI = (1.01–1.13) *p* = 0.027	ACE‐I/ARB, Albumin, Beta blocker, Bilirubin, Calcium, Chloride, Duration of primary HF diagnosis, GGT, Haemoglobin, History of anaemia, History of MI, History of PAD, History of PCI, ICD, Index event, NT‐proBNP at randomization, NYHA at baseline, Platelet, Heart rate, QTcF, Randomized while hospitalized, Systolic blood pressure, Urate
Docherty et al. (2025)	2940	22%	Patients with chronic HF	12 months	RCT	Significant	Baseline IL‐6 tertiles T1: ≤ 4.72 HR = 1 (ref) T2: 4.73–7.89 HR = 1.67 (1.30–2.14) *p* = 0.0001 T3: ≥ 7.90 HR = 3.04 (2.41–3.83) *p* < 0.001 IL‐6 per 1 U increase (log) 1.68 (1.53–1.84) *p* < 0.001	Significant	Baseline IL‐6 tertiles T1: ≤ 4.72 HR = 1 (ref) T2: 4.73–7.89 HR = 1.34 CI = (1.04–1.73) *p* = 0.025 T3: ≥ 7.90 HR = 1.80 CI = (1.41–2.31) *p* < 0.001 IL‐6 per 1 U increase (log) HR = 1.32 CI = (1.18–1.48) *p* < 0.001	Age, sex, systolic blood pressure, heart rate, ejection fraction, previous HF hospitalization, diabetes status, atrial fibrillation, ischemic aetiology, eGFR, randomized treatment (dapagliflozin), log hs‐TnT, and log NT‐proBNP
*HF hospitalization*										
Askevold et al. (2013)	1452	23%	Patients from CORONA trial, aged ≥ 60 years with chronic HF attributed to ischemic heart disease	955 days mean	RCT	Significant	1.40 (1.26–1.55) *p* < 0.001	Not significant	HR = 1.09 CI = (0.93–1.29) *p* = 0.270	LVEF, NYHA functional class, age, body mass index, diabetes mellitus, sex, intermittent claudication, heart rate, ApoB/Apo A‐1 ratio, estimated glomerular filtration rate, NT‐proBNP and hsCRP
Bielecka‐Dabrowa et al. (2013)	68	21%	Patients with dilated cardiomyopathy	60 months	Cohort, prospective	Not significant	NR	Not significant	NR	—
Markousis‐Mavrogenis et al. (2019)	2329	26%	Post doc analysis of the BIOSTAT‐CHF index cohort	21 months	Cohort, prospective	Not significant	1.06 (0.99–1.13) *p* = 0.069	Not significant	HR = 1.01 CI = (0.94–1.08) *p* = 0.833	Age, sex, BMI, eGFR, co‐morbidities, HF medications
Defilippi et al. (2023)	5050	24%	Post doc analysis of the VICTORIA trial	10.8 months mean	RCT	Significant	1.34 (1.27–1.40) *p* < 0.001	Not significant	HR = 1.05 CI = (0.98–1.12) *p* = 0.179	ACE‐I/ARB, Albumin, Beta blocker, Bilirubin, Calcium, Chloride, Duration of primary HF diagnosis, GGT, Haemoglobin, History of anaemia, History of MI, History of PAD, History of PCI, ICD, Index event, NT‐proBNP at randomization, NYHA at baseline, Platelet, Heart rate, QTcF, Randomized while hospitalized, Systolic blood pressure, Urate

Abbreviations: 6 MW, 6‐minute walk test; ACE‐I, angiotensin‐converting enzyme inhibitor; ADMA, asymmetric dimethylarginine; ANP, atrial natriuretic peptide; ApoB/ApoA‐1, apolipoprotein B/apolipoprotein A1 ratio; ARB, angiotensin receptor blocker; BMI, body mass index; BNP, brain natriuretic peptide; CT‐1, cardiotrophin‐1; hsCRP, high‐sensitivity C‐reactive protein; DCM, dilated cardiomyopathy; ET‐1, endothelin‐1; eGFR, estimated glomerular filtration rate; GGT, Gamma‐glutamyl transferase; HF, heart failure; hsCRP, high‐sensitivity C‐reactive protein; hs‐TnT, high‐sensitivity troponin T; HR, hazard ratio; IHD, ischemic heart disease; IL‐6, interleukin‐6; IL‐10, interleukin‐10; ICD, implantable cardioverter defibrillator; LVEF, left ventricular ejection fraction; MI, myocardial infarction; NE, norepinephrine; NGAL, neutrophil gelatinase‐associated lipocalin; NR, not reported; NT‐proBNP, N‐terminal pro‐B‐type natriuretic peptide; NYHA, New York Heart Association functional class; OR, odds ratio; PAD, peripheral artery disease; PCI, percutaneous coronary intervention; PNE, plasma norepinephrine; QTcF, Fridericia‐corrected QT interval; RCT, randomized controlled trial; RR, relative risk; sCD14, soluble CD14; SDMA, symmetric dimethylarginine; sTNF‐R1, soluble tumour necrosis factor receptor 1; sTNF‐R1, soluble tumour necrosis factor receptor 1; TNF‐alpha, tumour necrosis factor alpha.

#### 3.2.1 All‐Cause Mortality

Fourteen studies assessed all‐cause mortality across heterogeneous HF populations [5, 13, 15, 16, 17, 20, 21, 23, 24, 25, 26, 27, 28, 29]. IL‐6 was frequently associated with mortality in univariate analyses; however, findings were less consistent after multivariable adjustment. IL‐6 remained independently associated with mortality in approximately half of the studies, particularly those including larger cohorts [5, 20, 21, 24, 25], but lost significance in others after adjustment for clinical variables and established biomarkers such as natriuretic peptides and inflammatory markers (TNF‐alpha, sTNFR1, sTNFR2, hsCRP) [15, 16, 17, 23, 26, 27, 28, 29].

#### 3.2.2 Cardiovascular Death

Four large studies, including major RCTs (CORONA trial [16], DAPA‐HF [13], and VICTORIA trial [14]) and the BIOSTAT‐CHF [5] cohort also investigated CV death. A significant association between IL‐6 and CV death in univariate analysis was reported in all 4 studies and remained significant after multivariable adjustment in three studies but not in the CORONA trial (*n* = 1452). NT‐proBNP was part of the adjustment in three of the studies, including the CORONA trial, which was the only one to also adjust for hsCRP.

#### 3.2.3 HF Composites

Thirteen studies assessed HF composite outcomes, generally including cardiovascular death and HF re‐hospitalization [5, 13, 14, 16, 18, 19, 20, 22, 23, 27, 30, 31, 32]. IL‐6 showed a significant association in the majority of univariate analyses.

However, after multivariable adjustments, results were heterogeneous, with approximately half of the studies confirming independent prognostic value and the remainder showing loss of significance, particularly when natriuretic peptides were included in the models. Two studies further evaluated the incremental prognostic value of IL‐6 using discrimination and reclassification metrics. In the BIOSTAT‐CHF study by Markousis‐Mavrogenis et al. [5], addition of IL‐6 to the BIOSTAT risk model for the composite endpoint of all‐cause mortality and HF hospitalization yielded statistically significant but modest changes in prognostic performance (likelihood ratio test *p* = 0.002; NRI = −0.4%, *p* = 0.610; IDI = 0.2%, *p* = 0.031; Harrell's C = 0.713 vs. 0.715). Conversely, Docherty et al. [13] reported improved model performance for the composite outcome of worsening HF or all‐cause mortality after inclusion of IL‐6 alongside established biomarkers, including hs‐TnT and NT‐proBNP (NRI = 45.7%, 95% CI 33.7–55.6; IDI = 1.3%, 95% CI 0.2–2.4; *p* = 0.005 for AUC comparison), although the increase in AUC was relatively small (0.714 vs. 0.725).

#### 3.2.4 Hospitalization for HF

Hospitalization for HF was assessed as an outcome in four studies [5, 14, 16, 26], two of which found IL‐6 to be of significant prognostic value in univariate analyses, while the other two did not. After adjusting for main covariates such as NYHA class, LVEF, and NT‐proBNP, IL‐6 was no longer significant in all studies.

### 3.3 IL‐6 as a Pharmacodynamic Biomarker for Response to Therapies

We identified 43 RCTs and 16 observational studies (OBS) and non‐randomized intervention studies evaluating the effects of different treatment modalities on IL‐6 levels. Interventions included pharmacological therapies (23 RCTs and seven OBS), exercise (13 RCTs and one OBS), dietary supplements (five RCTs), device‐based treatments (one RCT and seven OBS), and combined interventions (including education intervention and balneotherapy, two RCTs).

Among the 23 RCTs assessing pharmacological therapies, eight investigated statins, three SGLT2 inhibitors, one beta‐blocker, two Angiotensin Receptor Blockers (ARBs), four levosimendan, two anti‐inflammatory drugs (colchicine and pentoxifylline), and one each Darbepoetin‐alfa, combination of two drug classes (Sacubitril/valsartan combined with vericiguat), and Chinese medicine. Four studies reported no significant change in IL‐6 [13, 33, 34, 35]. Sample sizes ranged from 12 to 2940 patients. Details on individual drugs and dosing regimens are provided in Table 2.

**TABLE 2:** Summary of literature on IL‐6 as pharmacodynamic biomarker in patients with HFrEF.

Author(s), year (References)	Study type	Treatment	Population (*n*)	Sample collection timepoint	Mean changes between groups after treatment	Mean changes before and after treatment (treatment group)	% changes before and after treatment (treatment group)
*Pharmacological interventions*							
Node et al. (2003)	RCT	Simvastatin	Treatment (*n* = 23) Placebo (*n* = 25)	3 months	−1 pg/mL*	−1.85 pg/mL	−47.4%*
Tousoulis et al. (2004)	RCT	4‐week atorvastatin treatment (10 mg/day)	Treatment (*n* = 19) Placebo (*n* = 19)	4 weeks	−0.8 pg/mL	−2.2 pg/mL	−28.2%*
Sola et al. (2006)	RCT	Atorvastatin	Treatment (*n* = 54) Placebo (*n* = 54)	12 months	−40 pg/mL*	−34 pg/mL	−20.4%*
Krum et al. (2007)	RCT	Rosuvastatin	Treatment (*n* = 31) Placebo (*n* = 38)	26 weeks	33.9 pmol/L^a^	27.90%^a^	27.9%^a^
Yamada et al. (2007)	RCT	Atorvastatin (10 mg/day)	Treatment (*n* = 19) Conventional treatment (*n* = 19)	6 months	0.1 pg/mL	−0.3 pg/mL	−11.5%
Bielecka‐Dabrowa et al. (2009)	RCT	Atorvastatin	Treatment (*n* = 41) Placebo (*n* = 27)	2 months	−12.82 pg/mL*	−7.75 pg/mL	−45.8%*
Duan et al. (2015)	RCT	Conventional treatment combined with atorvastatin	Treatment (*n* = 62) Conventional treatment (*n* = 61)	56 nights	−20 pg/mL*	−54.01 pg/mL	−63.3%*
Wang et al. (2020)	RCT	Atorvastatin (on the basis of routine therapy)	Treatment (*n* = 81) Routine symptomatic treatment (*n* = 81)	12 months	−2 pg/mL*	−6 pg/mL	−40.0%*
Sola et al. (2005)	Observational study	Statin (no dose description)	Treatment (*n* = 255) No statin (*n* = 191)	2 years	−40 pg/mL*	−38 pg/mL*	−22.2%*
Bielecka‐Dabrowa et al. (2013)	2 parallel cohorts	Atorvastatin (40 mg daily for 2 months followed by an individually matched dose of 10 or 20 mg/day)	Treatment (*n* = 22) No statin (*n* = 23)	5 years	−4.06 pg/mL*	NA	NA
Deftereos et al. (2014)	RCT	Colchicine	Treatment (*n* = 140) Placebo (*n* = 139)	6 months	−2.3 pg/mL*	−4.7 pg/mL^a^	−48.5%^a^
Avgeropoulou et al. (2005)	RCT	Levosimendan	Treatment (*n* = 15) Dobutamine (*n* = 14)	5 days	−8.3 pg/mL*	−3.8 pg/mL*	−44.2%*
Parissis et al. (2005)	RCT	Levosimendan	Treatment (*n* = 17) Placebo (*n* = 17)	48 h	−3.5 pg/mL	−3.9 pg/mL*	−24.1%*
Parissis et al. (2006)	RCT	Serial levosimendan infusions	Treatment (*n* = 17) Placebo (*n* = 8)	30 days	−7 pg/mL*	−2.3 pg/mL*	−17.6%*
Tasal et al. (2014)	RCT	Levosimendan infusion (a loading dose of 6 μg/kg for 10 min following a 24‐h infusion of 0.1 μg/kg/min)	A single dose (*n* = 16) Repeated (BL and at 1 and 3 months) (*n* = 13)	3 days, 6 months	Repeat dose vs. single does: 2.9 pg/mL (3 days), −10.83 pg/mL (6 months)*	singlE does: −7.8 pg/mL (3 days)*, 1.37 pg/mL (6 months). Repeat dose: −15.1 pg/mL (3 days)*, −19.3 pg/mL* (6 months)	single dose: −53.4% (3 days), −11.8% (6 months). Repeat dose: −60.9% (3 days), −77.8% (6 months)
Kyrzopoulos et al. (2005)	Cohort	Levosimendan infusion	Treatment (*n* = 12)	24 h, 72 h	NA	24 h vs. BL: −0.36 pg/mL; 72 h vs. 24 h: −0.07 pg/mL; 72 h vs. BL: −0.43 pg/mL*	24 h vs. BL: −19.8%; 72 h vs. 24 h: −4.8%; 72 h vs. BL: −23.6%
Ge et al. (2023)	RCT	Baseline treatments for left heart disease and PAH+ sacubitril/valsartan tablets + dapagliflozin tablets	Treatment (*n* = 60) Placebo (*n* = 60)	6 months	−3.18 pg/mL*	−15.57 pg/mL*	−46.2%*
Xie et al. (2024)	RCT	Dapaglifozin 10 mg/day	Treatment (*n* = 55) Placebo (*n* = 52)	3 months	−12.02 pg/mL*	−14.29 pg/mL*	−68.5%*
Docherty et al. (2025)	RCT	Dapaglifozin 10 mg/day	Treatment (*n* = 1488) Placebo (*n* = 1452)	12 months	NA	1.03 pg/mL	NA
Gotzmann et al. (2023)	A prospective, controlled, non‐randomized study	The SGLT‐2‐inhibitor empagliflozin (10 mg) in addition to standard medication	Treatment (*n* = 25) No empagliflozin (*n* = 25)	3 months	−5.3 pg/mL*	−8 pg/mL*	−36.9%*
Wood et al. (2024)	Observational study	SGLT2 inhibitors (either dapagliflozin or empagliflozin for a minimum of 12 months before muscle biopsy)	Treatment (*n* = 12) Standard of care (*n* = 16)	12 months	IL‐6 (serum): NA	IL‐6 (serum): NA	IL‐6 (serum): −64.10%*
					Muscle gene expression of IL‐6: NA	Muscle gene expression of IL‐6: NA	Muscle gene expression: −55.60%*
Wei et al. (2021)	RCT	Danshensu (30 mL/day injection)	Treatment (*n* = 55) Standard of Care (*n* = 30)	Unknown	−13.44 pg/mL*	NA	NA
Ogino et al. (2010)	RCT	ARB (added to baseline ACEi)	ARB + ACE‐i (*n* = 16) ACE‐i (*n* = 16)	16 weeks	−1.1 pg/mL	−1.3 pg/mL*	−32.5%
Tsutamoto et al. (2000)	RCT	Ang II Type 1 receptor (candesartan cilexetil)	Treatment (*n* = 14) Placebo (*n* = 9)	14 weeks	NA	−1.3 pg/mL*	−31.7%
Bahrmann et al. (2004)	RCT	Pentoxifylline	Treatment (*n* = 23) Placebo (*n* = 24)	6 months	−1 pg/mL^a^	−7 pg/mL	−58.3%
Kourea et al. (2008)	RCT	Darbepoetin‐alfa	Treatment (*n* = 21) Placebo (*n* = 20)	3 months	−8.2 pg/mL*	−4 pg/mL*	−38.1%*
Tian et al. (2021)	RCT	Metoprolol succinate (beta‐blocker) to control the heart rate (target rate: resting HR 50–60 beats/min)	Treatment (*n* = 41) Conventional intervention (*n* = 41)	3 months	−1.27 pg/mL*	−2.91 pg/mL*	−29.0%*
Jin et al. (2026)	RCT	Sacubitril valsartan sodium tablets and vericiguat	Treatment (*n* = 60) Control (sacubitril valsartan sodium tablets) (*n* = 60)	6 months	−5.26 pg/mL^a^	−10.93 pg/mL*	−54.0%*
Mayer et al. (2005)	A prospective observational study	Metoprolol succinate (beta‐blocker) (in increasing dosages (up to 190 mg/day) for the following 3 months)	Treatment (*n* = 21) General population for comparison of cytokine levels (*n* = 210)	3 months	NA	−4.4 pg/mL*	−49.44%*
Nessler et al. (2008)	Cohort	Arvedilol (beta‐blocker) Mean dose of carvedilol in the 3rd month was 23.0 ± 13.5 mg/day and after 12 months 25.8 ± 15.2 mg/day	Treatment (*n* = 86)	3 and 12 months	NA	0–12 months: −4.1 pg/mL*	−43.60%*
*Exercise*							
Adamopoulos et al. (2002)	RCT	Exercise Training (a 12‐week, home‐based, bicycle exercise training programme 5 days per week, 30 min per day, or asked to avoid exercise (detraining) in a randomized, crossover design)	Aerobic exercise (*n* = 24)	12 weeks	↓* (CHF > health control at BL)	Between training and BL: ↓*, between training and detraining: ↓*	
Gielen et al. (2003)	RCT	Exercise training	Aerobic exercise (*n* = 10) Sedentary lifestyle (*n* = 10) Stable CHF	6 months	0.3 pg/mL	NA	
					−14.2 relative units*	−30 pg/mL*	−42.1%
Kobayashi et al. (2003)	RCT	Aerobic exercise (supervised cycle ergometer training for 2–3 days per week in two 15‐min sessions per day for 3 months)	Aerobic exercise (*n* = 14) No exercises (*n* = 14)	3 months	−0.73 pg/mL	−1.22 pg/mL	−37.9%
Niebauer et al. (2005)	RCT	Aerobic exercise (at least 5 days a week of a combination of callisthenics (the Canadian airforce XBX programme 9 min) and bicycle ergometry for 20 min a day, performed at home)	Aerobic exercise (*n* = 18) No exercises (*n* = 9)	8 weeks	20.5 pg/mL	−0.5 pg/mL	−1.4%
Pullen et al. (2008)	RCT	Yoga	Yoga (*n* = 9) Control (*n* = 10)	2 months	−5.3 mg/dL*	−3.9 mg/dL*	−22.3%*
Pullen et al. (2010)	RCT	Supervised hospital‐based yoga	Yoga (*n* = 21) Control (*n* = 19)	2 months	−2.01 pg/mL*	−3.57 pg/mL*	−18.9%*
Byrkjeland et al. (2011)	RCT	High intensity exercise training	Aerobic exercise (*n* = 40) No exercises (*n* = 40)	4 months, 12 months follow‐up	4 months: −1.3 pg/mL 12 months: −0.79 pg/mL	4 months vs. BL: 0.72 pg/mL^a^ 12 months vs. BL: −0.18 pg/mL^a^	4 months vs. BL: 20.9% 12 months vs. BL: −5.2%
Feiereisen et al. (2013)	RCT	Endurance training (ET): Bicycle and treadmill, progressing from 60% to 75% VO_2_ peak Strength training (ST): 10 resistance exercises, starting at 60% 1RM and increasing to 75% Combined endurance and strength training (CT): 20 min endurance + 20 min strength training (5 machines), same progression	ET (*n* = 15): 40 min Bicycle and treadmill, progressing from 60% to 75% VO_2_ peak ST (*n* = 15): 10 resistance exercises during 40 min, starting at 60% 1RM and increasing to 75% CT (*n* = 15): 20 min endurance + 20 min strength training (5 machines), same progression No exercise (*n* = 15)	48 h	CT vs. Control: −0.64 pg/mL* ET vs. Control: −0.78 pg/mL* ST vs. Control: −0.82 pg/mL*	CT: −1.35 pg/mL* ET: −0.84 pg/mL* ST: −1.29 pg/mL*	CT: −28.5%* ET: −20.5%* ST: −34.4%*
Zeinab Helmy et al. (2013)	RCT	Resisted exercise training (40–50 min, resisted exercises, 3 times/week)	Resisted exercise (*n* = 20) No exercises (*n* = 20)	12 weeks	−0.07 pg/mL	−0.03 pg/mL	−0.4%
Eleuteri et al. (2013)	RCT	Aerobic exercise training	Aerobic exercise (*n* = 11) No exercises (*n* = 10)	3 months	0.9 pg/mL^a^	−0.4 pg/mL*	−17.4%
de Meirelles et al. (2014)	RCT	Combination exercise training (30 min of moderate‐intensity treadmill exercise, followed by resistance and stretching exercises, performed three times a week)	Combination exercise training (*n* = 15) No exercises (*n* = 15)	6 months	−0.5 pg/mL	−0.5 pg/mL	−11.1%
Fernandes‐Silva et al. (2017)	RCT	Aerobic exercise (the aerobic interval training comprised alternated intensity. Three supervised sessions a week, during 12 weeks. Each exercise session was performed in a cycloergometer during 30 min, added to 5‐min warming and cool‐down respectively)	Aerobic exercise (*n* = 28) No exercises (*n* = 16)	12 weeks	NA	−0.1 pg/mL^a^	NA
Omidi et al. (2020)	RCT	Aerobic exercises	Aerobic exercises (*n* = 10) No exercises (*n* = 10)	8 weeks	−2.02 (unit unknown)^a^	−2.03 (unit unknown)*	−54.8%*
Conraads et al. (2002)	Non‐randomized trial	Exercise training	Exercise (*n* = 23) No exercises (*n* = 18)	4 months	NA	−0.8 pg/mL	−38.1%
*Dietary supplements*							
Nodari et al. (2009)	RCT	PUFA 1 g/day	PUFA 1 g/day (*n* = 22) Placebo (*n* = 22)	6 months	−1.55 pg/mL*	−6.7 pg/mL*	−68.4%*
Zhao et al. (2009)	RCT	2 g/day of n‐3 PUFA	2 g/day of n‐3 PUFA (*n* = 38) Placebo (*n* = 37)	3 months	−4.9 pg/mL*	−3.6 pg/mL*	−31.3%*
Nodari et al. (2011)	RCT	PUFA 1–5 g/day	PUFA 2 g/day (*n* = 67) Placebo (*n* = 66)	12 months	−7.67 pg/mL*	−7.47 pg/mL*	−67.9%*
Moertl et al. (2011)	RCT	PUFA 1 and 4 g/day	PUFA 1 or 4 g/day (*n* = 27) Placebo (*n* = 16)	3 months	hs‐IL‐6: 1 g/day n3‐PUFA vs. Control: −2.03 pg/mL^a^ 4 g/day n3‐PUFA vs. Control: −2.99 pg/mL^a^	hs‐IL‐6: 1 g/day n3‐PUFA: −2.5 pg/mL 4 g/day n3‐PUFA: −2.3 pg/mL*	hs‐IL‐6: 1 g/day n3‐PUFA: −61.0% 4 g/day n3‐PUFA: −76.7%*
Zhang et al. (2022)	RCT	Enteral nutrition support therapy (intact protein enteral nutrition + dietary fibre + monounsaturated fatty acids and fructose [diabetes])	Enteral nutrition support therapy (*n* = 40) Restricted salt and water intake (*n* = 40)	2 weeks	−4.11 pg/mL*	−9.96 pg/mL*	−54.0%*
*Devices intervention*							
Rubaj et al. (2006)	Cross‐over study	Right ventricular pacing	Cross‐over study (*n* = 28)	BiVP 48 h	−7.1 pg/mL*	NA	NA
Lappegård et al. (2006)	Cohort	CRT	*n* = 8	6 months	NA	−1.15 pg/mL*	−45.1%*
Seifert et al. (2007)	Cohort	CRT	*n* = 22	12 months	NA	NS	NA
Boriani et al. (2006)	Cohort	CRT	*n* = 32	12 months	NA	NS	NA
Theodorakis et al. (2006)	Cohort	CRT	*n* = 20	3 months after pacing therapy, and after a subsequent 3‐month period of no pacing	NA	BL: 8.9 ± 2.5 pg/mL Following 3 Months of Biventricular Pacing: 4.7 ± 1.3 pg/mL* After 3 Months of Intrinsic Rhythm: 3.9 ± 1.1 pg/mL*	Following 3 months of biventricular pacing: −47.2%* After 3 months of intrinsic rhythm: −56.2%
Przybyła et al. (2011)	Observational study	CRT	*n* = 38	3 months	NA	−4.7 pg/mL*	−46.5%*
Caruso et al. (2012)	Observational prospective	Continuous‐flow LVADs (DeBakey, Incor, HeartMate II)	LVADs (*n* = 23) Stable ESHF patients (*n* = 22), healthy controls (*n* = 20)	30 days	NA	Pre‐implant: 12.8 pg/mL (3.6, 37.2) 1 day: 376.5 pg/mL (177.4, 590.4)* 7 days: 61.0 pg/mL (30.3, 123.3)* 30 days: 20.2 pg/mL (8.2, 30.6)*	1 day: +2841.4%* 7 days: +376.5% 30 days: +57.8%
Stieger et al. (2018)	Observational study	Internal cardioversion	Internal cardioversion (*n* = 22) External cardioversion (*n* = 42)	8 h	NA	Internal cardioversion: 4.7 pg/mL* External cardioversion: 1.5 pg/mL	Internal cardioversion: 77.1%* External cardioversion: 34.1%
*Other interventions*							
Oyama Jet al. (2013)	RCT	Balneotherapy (half‐body immersion in 40°C hot spring, 10 min/day, 5 days/week, 2 weeks)	Balneotherapy (*n* = 16) Daily shower (*n* = 16)	2 weeks	−1.9 pg/mL*	−1.55 pg/mL*	−38.3%*
Tian et al. (2024)	RCT	Education intervention (emotional stability, dietary, activity, medication)	Education intervention (*n* = 30) Conventional intervention (*n* = 30)	Unknown	−34.69 pg/mL*	−61.22 pg/mL*	−47.4%*

*Note:* IL‐6 units were harmonized to pg/mL whenever possible. For Pullen et al. (2008), the original unit (mg/dL) was retained because it was consistently reported in the source publication and no alternative unit could be verified.

Abbreviations: BL, baseline; CHF, chronic heart failure; CRT, cardiac resynchronization therapy; NS, non‐significant.

^a^ NA, not tested.

* *p* < 0.05.

#### 3.3.1 Statins

Eight RCTS [33, 34, 36, 37, 38, 39, 40, 41] and two OBS [42, 43] evaluated statins (atorvastatin, rosuvastatin, simvastatin). Overall, statin therapy generally reduced IL‐6 levels, although effects varied by agent, dose and duration. Atorvastatin trials reported IL‐6 reductions ranging from 20.4% [41] to 63.3% [37] depending on dose and follow‐up; simvastatin showed a 47.4% decrease in a small RCT (*n* = 51) [40], while a rosuvastatin trial found no significant effect [33]. Observational studies also support lower IL‐6 with statin use but are limited by potential confounding [42, 43].

#### 3.3.2 Sodium Glucose Transporter 2 (SGLT‐2) Inhibitor

Three RCTs [13, 44, 45], one non‐randomized intervention study [46] and one OBS [47] assessed SGLT2 inhibitor, mostly dapagliflozin, effect on IL‐6, yielding inconsistent findings. Two small RCTs [44, 45] and a non‐randomized study [46] reported significant short‐term IL‐6 reductions whereas the large DAPA‐HF trial (*n* = 2940) observed no IL‐6 reduction over 12 months [13]. One comparative OBS also reported decreased downstream muscle IL‐6 signalling and lower serum IL‐6 level with SGLT2i inhibitor therapy (dapagliflozin or empagliflozin) [47].

#### 3.3.3 Beta‐Blocker

One RCT [48] found that metoprolol succinate used to actively control heart rate significantly reduced IL‐6 levels and other inflammatory markers, while also improving LVEF and cardiac output. Two OBS evaluated beta‐blocker on IL‐6 levels: metoprolol‐succinate was associated with a 49.4% significant IL‐6 reduction at 3 months (*n* = 21) [49] and carvedilol showed a significant IL‐6 decrease after 12‐month treatment, but not at three‐month follow‐up (*n* = 86) [50].

#### 3.3.4 Angiotensin II Receptor Blockers (ARBs)

In a 16‐week crossover RCT, addition of losartan (100 mg/day [*n* = 13] or 50 mg/day [*n* = 3]) to ACE‐inhibitor therapy significantly decreased the serum levels of IL‐6 from baseline compared with ACE‐inhibitor therapy alone [51]. Similarly, 14 weeks of candesartan cilexetil decreased IL‐6 by 31.7% in 14 congestive HF patients, while the control group (*n* = 9) showed no significant changes [52].

#### 3.3.5 Levosimendan Infusions

One OBS [53] and four RCTs [54, 55, 56, 57] evaluated the effect of serial levosimendan infusions in patients with severe decompensated HFrEF. The RCTs reported IL‐6 reductions of 24.1% within 48 h [54], 44.2% after 5 days [56], and 17.6% after 30 days [55]. Another RCT found marked declines at 72 h after single (−53.4%) and repeated (−60.9%) dosing and larger long‐term reductions only with repeated infusions [57].

#### 3.3.6 Other Pharmacological Interventions

Small trials reported IL‐6 significant reductions with darbepoetin‐α (−38.1% at 3 months) [58] and with danshensu injection [59]. In a larger RCT, colchicine (0.5 mg twice daily) lowered IL‐6 by 48.5% over 6 months without improving functional status or reducing HF events [60]. Pentoxifylline showed no significant IL‐6 change after 6 months [35]. As for combination therapy, sacubitril valsartan sodium tablets combined with vericiguat (angiotensin receptor–neprilysin inhibitor and soluble guanylate cyclase stimulator) [61] reduced IL‐6 levels more than sacubitril/valsartan alone after 6 months in CHF patients.

#### 3.3.7 Exercise

Exercise protocols have shown varying effects on IL‐6 levels. Two resistance‐training RCTs (3–4 months) showed either no [62] or a non‐significant decrease of IL‐6 [63]. Among nine small aerobic RCTs (*n* = 20–80), two reported significant IL‐6 reductions [64, 65], contrary to the other seven studies [66, 67, 68, 69, 70, 71, 72]. Local skeletal muscle IL‐6 mRNA expression was reduced by 42% in aerobic training patients [66]. A combined aerobic and resistance training over 6 months did not significantly impact IL‐6 levels [73]. Two small RCTs in mainly African American patients showed IL‐6 reductions and improved exercise tolerance with twice‐weekly yoga [74, 75].

#### 3.3.8 Dietary Supplements

Polyunsaturated fatty acids (PUFAs) have varied effects on IL‐6 concentrations among studies, with two RCTs showing no significant reduction [76, 77] and two others showing significant reduction [78, 79]. An additional RCT (*n* = 80) showed that enteral dietary support therapy reduced IL‐6 level significantly after 2 weeks of treatment in moderate and severe HF patients [80].

#### 3.3.9 Devices Intervention

The effect of device intervention included five studies of cardiac resynchronization therapy [81, 82, 83, 84, 85]. The studies showed variable changes in IL‐6 levels, with only one reporting a significant reduction [84]. Additionally, a small cross‐over study demonstrated a decrease in IL‐6 after switching for 48 h from right to bi‐ventricular pacing [86].

A prospective OBS found a large rise of IL‐6 levels within 24 h after left ventricular assist device (LVAD) implantation in end‐stage HF patients, returning to levels comparable to pre‐implant values after 1 month [87]. An OBS reported a significant increase in IL‐6 in patients who underwent internal electrical cardioversion using implantable cardioverter‐defibrillator [88].

#### 3.3.10 Other Interventions

A RCT showed that the educational intervention reduced IL‐6 levels significantly [89]. Balneotherapy (half‐body immersion in 40°C hot spring, 10 min/day, 5 days/week) was also found to reduce IL‐6 after 2 weeks [90].

### 3.4 IL‐6 Changes and Clinical Benefit

In CORONA trial, rosuvastatin showed no effect on either IL‐6 levels or clinical outcomes, indicating a neutral profile on both biomarker modulation and prognostic benefit (Table S6). Among SGLT2 inhibitors, heterogeneous patterns were observed. Dapagliflozin in the largest cohort of HF patients (*n* = 2940) showed a robust improvement in cardiovascular outcomes despite no significant change in IL‐6 levels, highlighting a clear dissociation between IL‐6 modulation and therapeutic efficacy. For β‐blockers (carvedilol, metoprolol) and renin–angiotensin system inhibitors (losartan, candesartan cilexetil), small studies reported reductions in IL‐6, while the clinical benefits of these drug classes are observed from broader HFrEF trial evidence. A similar pattern observed was associated with reductions in IL‐6 and improved outcomes, but this does not establish that IL‐6 reduction mediates or predicts clinical benefit.

Levosimendan represents an intermediate case, with reductions in IL‐6 but inconsistent effects on clinical outcomes across studies, limiting the interpretability of the biomarker‐response relationship. Among other therapies, several discordant patterns emerged. Cardiac resynchronization therapy and polyunsaturated fatty acids were both associated with clinical benefits despite no consistent changes in IL‐6 levels. Conversely, darbepoetin alfa and colchicine reduced IL‐6 without translating into improved outcomes, indicating a lack of correspondence between biomarker modulation and treatment effect. Finally, pentoxifylline showed no clear changes in IL‐6 and limited clinical evidence of benefit, based only on small and exploratory studies. Taken together, these heterogeneous and often discordant patterns indicate that treatment‐induced changes in IL‐6 were inconsistently concordant with clinical benefit across interventions.

### 3.5 IL‐6 as a Predictive Biomarker to Identify Responders to Therapy

Three studies evaluated the predictive role of IL‐6, and none found a significant association.

In a secondary analysis of the VICTORIA sub study (*n* = 5050), Defilippi et al. [14] examined whether baseline IL‐6 predicted response to 16 weeks of vericiguat. Although IL‐6 was prognostic, no predictive value was identified contrary to NT‐proBNP and high‐sensitivity Troponin T (hs‐cTnT) [14].

Sunman et al. classified 32 of 44 CRT‐treated patients as responders based on a decrease in Left Ventricular End‐Systolic Volume (LVESV) ≥ 15% at 12 months [91]. IL‐6 decreased significantly in responders, but baseline values did not predict CRT response, contrary to BNP and oxidized Low‐density Lipoprotein (oxLDL).

In the DAPA‐HF trial (*n* = 2940), dapagliflozin reduced cardiovascular and HF outcomes similarly across all IL‐6 levels. Baseline IL‐6 did not predict differential treatment benefit, and the effects were consistent across IL‐6 concentrations [13].

## 4 Discussion

This review shows that circulating IL‐6 in HFrEF is biologically informative but context‐dependent. Higher IL‐6 concentrations generally identify patients with greater inflammatory burden and more advanced disease, but incremental prognostic value beyond established HF biomarkers is limited. Several interventions reduce IL‐6, but the relationship between IL‐6 reduction and clinical benefit is inconsistent. No evidence supports IL‐6 as a predictive biomarker for selecting therapy. Therefore, IL‐6 may serve as a response biomarker in selected contexts, but current evidence does not support IL‐6 as a universal surrogate measure for clinical outcome improvement in HFrEF.

### 4.1 Prognostic Value: IL‐6 Reflects Risk, but Not Enough for Clinical Action

The prognostic signal of IL‐6 appears strongest for mortality and weaker for HF hospitalization. However, the attenuation after adjustment for natriuretic peptides, hsCRP, renal function, NYHA class, and other markers suggests that IL‐6 often captures global disease severity and systemic inflammatory burden, rather than providing a distinct, decision‐changing risk signal. For example, the CORONA trial included both hsCRP and NT‐proBNP within the same model, making it difficult to distinguish their individual contributions [16]. Though the finding is consistent with the fact that CRP is an acute phase protein produced mostly by the liver under stimulation by IL‐6 and other cytokines.

Despite its limited standalone performance, IL‐6 may retain value for inflammatory phenotyping. The recent ODYSSEY OUTCOMES post‐ACS analysis [92] provides an instructive example: hsCRP and IL‐6 were only moderately correlated, and patients had significantly higher risks of MACE and death only when both markers were elevated, not when either marker was elevated alone. Moreover, although hsCRP was the stronger predictor of MACE, both hsCRP and IL‐6 independently predicted all‐cause mortality after mutual adjustment. These observations should be extrapolated cautiously because ACS and HFrEF represent different clinical contexts. However, they reinforce the concept that IL‐6 and hsCRP are related but non‐redundant inflammatory markers. In HFrEF, where IL‐6 is associated with disease severity and mortality but often loses incremental value after adjustment for NT‐proBNP and other established markers, IL‐6 is unlikely to function as a routine standalone risk marker. Consistent with this interpretation, the few studies that formally assessed its incremental prognostic performance beyond conventional risk models reported heterogeneous findings, with statistically significant improvements in some discrimination and reclassification metrics but generally modest gains in overall predictive performance. Therefore, the clinical question should not be whether IL‐6 replaces established biomarkers, but whether IL‐6‐axis markers can refine inflammatory phenotyping in selected patients or trial populations. Its more plausible role may be in multimarker strategies that combine myocardial stress, injury, renal dysfunction, systemic inflammation, and IL‐6‐axis activity to identify biologically distinct high‐risk subgroups.

### 4.2 Interpretation of IL‐6 Changes: Three Different Meanings

A key finding of this review is that IL‐6 reduction has different meanings depending on the intervention. A significant biomarker change after treatment may reflect pathway engagement, system response or potential surrogacy; while these concepts are not equivalent.

For interventions that are designed to modulate IL‐6 or upstream inflammatory pathways, treatment‐related changes in IL‐6 may represent a pharmacodynamic biomarker consistent with pathway engagement. For interventions without a primary anti‐inflammatory target, IL‐6 reduction is better interpreted as a downstream pharmacodynamic response reflecting broader biological effects rather than evidence of direct target engagement. In either setting, a decrease in IL‐6 indicates that a specific inflammatory pathway has been modulated by treatment, but it does not establish improvement in HF pathophysiology or clinical outcomes. Besides, interventions differ in their effects on IL‐6 due to differences in mechanisms and tissue distribution. For example, statins exert anti‐inflammatory effects by inhibiting isoprenoid‐dependent prenylation and downstream IL‐6–inducing pathways [93], the extent to which differences in membrane permeability and tissue distribution determine cytokine suppression across HF phenotypes remains unclear.

By contrast, for therapies without a primary anti‐inflammatory mechanism, IL‐6 reduction may reflect improvement in the broader HF disease state, including reduced congestion, neurohormonal activation, tissue stress or systemic inflammatory activation. These two interpretations should not be confused. Colchicine illustrates the first situation, which is that IL‐6 may fall as a pharmacodynamic signature of anti‐inflammatory activity, yet this does not necessarily translate into HF benefit. Beta‐blockers, ARBs or other disease‐modifying therapies may illustrate the second situation, where IL‐6 reduction may reflect improvement in the patient's biological state rather than direct cytokine targeting.

The pharmacodynamic evidence supports IL‐6 as an intervention‐specific biomarker rather than a general treatment‐response marker. IL‐6 reduction was more consistent with interventions expected to affect inflammation or acute hemodynamics, such as statins and levosimendan, but less consistent with SGLT2 inhibitors, exercise, nutritional interventions and device therapies. Therefore, IL‐6 should not be interpreted uniformly across interventions. The same numerical change in IL‐6 may have different biological meanings depending on whether the intervention directly suppresses inflammatory signalling, improves HF physiology, alters skeletal muscle biology, or changes congestion and tissue perfusion.

### 4.3 IL‐6 Reduction Is Not Supported as a Surrogate Measure for Clinical Outcome Improvement in HFrEF

The evidence reviewed here does not support IL‐6 reduction as such a surrogate in HFrEF. A surrogate measure should reliably predict clinical benefit within the intended disease indication and context of use. Several therapies with proven or plausible clinical benefit do not consistently reduce IL‐6, whereas some interventions reduce IL‐6 without clear improvement in HF outcomes. Moreover, biomarker and outcome evidence often come from different studies, limiting causal interpretation. This discordance indicates that IL‐6 lowering cannot be used as a universal substitute for clinical endpoints in HFrEF trials. At most, IL‐6 may be considered a response biomarker in selected contexts, particularly when interpreted alongside the mechanism of action and other markers of HF biology. If IL‐6 is used in a surrogate context, current evidence supports its inclusion only as one component of a multimarker panel rather than as a standalone universal surrogate measure.

### 4.4 IL‐6 Is Not a Predictive Biomarker for Treatment Selection

The limited available evidence does not support baseline IL‐6 as a predictor of treatment response in HFrEF. In DAPA‐HF [13] and VICTORIA, baseline IL‐6 was prognostic but did not identify patients with different treatment benefits. This distinction is important because a biomarker can identify high‐risk patients without identifying patients more likely to respond to a specific therapy.

### 4.5 Why Isolated Circulating IL‐6 May Be Insufficient: The IL‐6 Signalling Axis

The limited clinical utility of isolated IL‐6 measurement is biologically plausible. Circulating IL‐6 alone is not equivalent to IL‐6 pathway activation. Briefly, the biological activity of IL‐6 is determined by two distinct signalling pathways: classic signalling via membrane‐bound IL‐6R (primarily anti‐inflammatory and regenerative) and trans‐signalling via sIL‐6R (predominantly pro‐inflammatory) [94]. The reason this distinction is important in HFrEF is that cardiac myocytes lack IL‐6 receptors, so that the only way that IL‐6 can signal to the heart is by binding soluble IL‐6 receptors (sIL‐6R) and then the complex of IL‐6/sIL‐6R binds to gp130 receptors on cardiac myocytes. The ratio of IL‐6:sIL‐6R complexes to inactive IL‐6:sIL‐6R:sgp130 (which acts as a decoy soluble receptor) complexes is associated with cardiovascular risk, demonstrating that measuring both components provides clinically relevant prognosis [95]. Most IL‐6 remains free rather than complexed with sIL‐6R under physiological conditions, allowing simultaneous classic and trans‐signalling. However, the relative balance between free IL‐6, IL‐6:sIL‐6R complexes, and inhibitory IL‐6:sIL‐6R:sgp130 complexes determines which signalling pathway predominates [96]. Isolated IL‐6 measurements fail to capture the full spectrum of biological activity because they do not account for the availability of sIL‐6R to drive trans‐signalling or the presence of sgp130 to inhibit it. Therefore, IL‐6 may be a marker of systemic inflammation, not a cardiac‐specific marker of IL‐6‐axis activity. This biological limitation may help explain why IL‐6 is consistently associated with disease severity but has not performed reliably as a predictive biomarker or as a standalone pharmacodynamic/surrogate marker.

### 4.6 Assay and Treatment Limitations

Several factors limit interpretation of IL‐6 across studies. First, substantial heterogeneity exists in pre‐analytical procedures, including differences in specimen matrix (serum vs. plasma), anticoagulant use, as well as sample handling and processing conditions (Table S3). These factors may influence measured IL‐6 concentrations and contribute to inter‐study variability independent of underlying biological differences. Analytical heterogeneity was also assumed to be significant, including differences in assay sensitivity, measurement range, calibration procedures, antibody specificity, precision and platform performance. Consequently, absolute IL‐6 concentrations and thresholds should not be considered interchangeable across assays. Importantly, a statistically significant change may still fall within analytical variability or lack biological relevance, particularly when assay precision and limits of quantification are incompletely reported. In conclusion, the most reliable evidence supports the use of the direction of IL‐6 changes within individual studies rather than the comparison of absolute IL‐6 concentrations across studies.

Second, background HFrEF therapy changed markedly across eras. Earlier cohorts were often treated with narrower neurohormonal blockade including ACEi and β‐blockers, whereas current guideline‐directed medical therapy (GDMT) for HFrEF, such as ARNI/ACEi/ARB, beta‐blockers, MRAs and SGLT2 inhibitors, may reduce inflammatory burden directly or indirectly through improved hemodynamics, neurohormonal modulation, decongestion or metabolic effects [97, 98]. Because these therapies may affect systemic inflammation, IL‐6 distributions and prognostic associations may not be stable across treatment development. Lastly, IL‐6 is a non‐specific inflammatory marker and may be elevated due to comorbidities that are common in HFrEF patients such as infections, autoimmune diseases, diabetes, COPD and cancer [99]. Differences in comorbidity across cohorts or trials may therefore confound both baseline IL‐6 level and their associations with outcomes.

Together, these factors limit cross‐study comparability and highlight the need for standardized assays, transparent analytical reporting, and clearly defined clinical contexts of use.

### 4.7 Strengths

Most prior reviews addressed specific features of IL‐6 as circulating biomarkers. We comprehensively reviewed IL‐6 according to three main uses of the biomarkers: prognostic, pharmacodynamic and predictive. Publications on prognostic use of IL‐6 were assessed with QUIPS evaluation. An AI‐based tool leveraging a LLM was developed ad hoc to check the full text of all publications for compliance with inclusion/exclusion criteria.

### 4.8 Limitations

Several limitations must be acknowledged, mostly inherent to the studies reviewed. First, publication bias cannot be excluded, although risk assessment across prognostic studies showed predominantly low‐to‐moderate bias, with only five studies rated moderate‐to‐high.

Secondly, substantial heterogeneity in study design, populations and IL‐6 assays prevented meta‐analysis and limits the generalizability [100], though overall trends were consistent across assays.

Finally, different studies applied different LVEF thresholds for HFrEF (Table S7): 60 of the 80 included studies (75.0%) used an LVEF threshold of ≤ 40% or lower, and 73 studies (91.3%) used a threshold of ≤ 45% or lower. Only 5 studies (6.3%) enrolled patients using a threshold extending up to < 50%. This made it impossible to perform sensitivity analysis of the specific LVEF groups.

## 5 Conclusion

While there is evidence for an association between IL‐6 and adverse clinical outcomes, current evidence suggests that IL‐6 may be better suited for inflammatory and biological phenotyping, as well as selected mechanistic and pharmacodynamic research applications than for standalone clinical decision making in HFrEF. Evidence does not support IL‐6 as a predictive biomarker for treatment response, as a standalone prognostic marker for routine risk stratification, or for monitoring of therapeutic efficacy. Treatment‐induced IL‐6 changes also do not consistently track with clinical outcome improvement. For drug development, IL‐6 may be useful as a pharmacodynamic biomarker reflecting target or pathway engagement when an intervention is expected to modulate inflammatory pathways, but IL‐6 reduction should not be interpreted as evidence of clinical efficacy. For future studies, IL‐6 should be evaluated in prespecified contexts of use, ideally as part of a multimarker panel incorporating natriuretic peptides, myocardial injury markers, renal markers, hsCRP, and IL‐6‐axis components such as sIL‐6R and sgp130.

## Author Contributions

**Yongxin Ye:** writing – original draft, project administration, data curation, investigation, validation, visualization. **Martina Colombo:** writing – original draft, project administration, data curation, investigation, validation, visualization. **Jennifer Meessen:** writing – review and editing, investigation, data curation, methodology, conceptualization. **Benoit Tyl:** writing – review and editing, investigation, data curation, methodology, conceptualization. **Deborah Novelli:** writing – review and editing, investigation, data curation. **Negin Hajizadeh:** writing – review and editing, investigation, data curation, methodology, conceptualization. **Hans‐Peter Brunner‐La Rocca:** writing – review and editing, funding acquisition, conceptualization. **Adriaan Voors:** writing – review and editing, conceptualization. **Sara Klas:** writing – review and editing, investigation, data curation. **Stephan Schwers:** writing – review and editing, investigation, data curation. **Bjarke Follin:** writing – review and editing, investigation, data curation, methodology, conceptualization. **Roberto Latini:** supervision, writing – original draft, validation, methodology, conceptualization.

## Funding

This research is part of the Individualised Care from Early Risk of Cardiovascular Disease to Established Heart Failure (iCARE4CVD) project. iCARE4CVD has received funding from the Innovative Health Initiative Joint Undertaking (IHI JU) and BreakthroughT1D under grant agreement No. 101112022. The JU received support from the European Union's Horizon Europe research and innovation programme and COCIR, EFPIA, Vaccines Europe, EuropaBio and MedTech Europe. Views and opinions expressed are however those of the author(s) only and do not necessarily reflect those of the aforementioned parties. Neither of the aforementioned parties can be held responsible for them.

## Ethics Statement

The authors have nothing to report.

## Conflicts of Interest

Y.Y. and B.F. are full‐time employees of Novo Nordisk. B.T., S.S. and N.H. are full‐time employees of Bayer. S.K. is a full‐time employee in Evotec.

## Supporting information


**Table S1:** Exclusion/inclusion criteria.
**Table S2:** Description of the three biomarker categories.
**Table S3:** Pre‐analytical and analytical characteristics of IL‐6 measurement across included studies.
**Table S4:** Summary of literature on IL‐6 as prognostic biomarker in patients with heart failure with reduced ejection fraction (HFrEF).
**Table S5:** Risk of bias assessment of included studies using the QUIPS tool.
**Table S6:** Comparison between treatment‐induced IL‐6 changes and available clinical outcome evidence.
**Table S7:** Distribution of LVEF% cutoffs in the 80 selected publications.
**Figure S1:** Summary of risk of bias.

## Data Availability

The data that support the findings of this study are available from the corresponding author upon reasonable request.
