# Supplementary material for: Why Circulating Interleukin‐6 Is Not Yet a Standalone Clinically Actionable Biomarker in HFrEF: A Context‐of‐Use Review
Source: Eur J Clin Invest. 2026 Oct 17;56(10):e70261. doi: 10.1111/eci.70261 (PMC13675830; doi:10.1111/eci.70261)
Supplement: Supplementary file 1 [file ECI-56-e70261-s001.docx]

**Supplementary Appendix**

**Why Circulating Interleukin-6 Is Not Yet a Standalone Clinically Actionable Biomarker in HFrEF: A Context-of-Use Review**

Yongxin Ye^1*^, Martina Colombo^2*^, Jennifer Meessen^2^, Benoit Tyl^3^, Deborah Novelli^2^, Negin Hajizadeh^4^*,* Hans-Peter Brunner-La Rocca^5,6^, Adriaan Voors^7^, Sara Klas^8^, Stephan Schwers^9^, Bjarke Follin^1^, Roberto Latini^2^.

^*^shared first authors

^1^ Novo Nordisk A/S, Søborg, Denmark;

^2^ Department of Acute Brain and Cardiovascular Injury, Istituto di Ricerche Farmacologiche Mario Negri IRCCS, Milan, Italy;

^3^ Bayer HealthCare SAS, La Garenne Colombes, France;

^4^ Bayer Espana AG, Madrid, Spain;

^5^ Cardiovascular Research Institute Maastricht (CARIM), Maastricht University, PO Box 616, 6200MD Maastricht, The Netherlands;

^6^ Department of Cardiology, Maastricht University, Medical Center MUMC+, PO Box 5800, 6202AZ Maastricht, The Netherlands;

^7^ University of Groningen, Department of Cardiology, University Medical Center Groningen, University of Groningen, The Netherlands;

^8^ Evotec International, GmbH, Hamburg, Germany;

^9^ Bayer AG, Wuppertal, Germany.

**Supplementary methods**

**PICO framework**

Population: heart failure with reduced ejection refraction

Intervention: Biomarkers

Comparison: Any

Outcome: Any

**Search strategy and selection criteria**

We searched MEDLINE and EMBASE for studies published up to June 2026 using an ad hoc designed search string (see below). Our review included original studies in HFrEF published in English. We employed the population, intervention (biomarkers), comparison, and outcome (PICO) framework (1) as the criteria for inclusion and exclusion of publications (Table S1). Additionally, relevant narrative reviews, systematic reviews, and meta-analyses were used as supplementary sources to identify studies not captured by the main search strategy. The references listed in these reviews were manually screened, and those meeting the inclusion criteria were added to the pool of publications.

*PubMed search string*

(Inhibitor* Interleukin-6[Title/Abstract] OR IL-6 Inhibitor*[Title/Abstract] OR IL6[Title/Abstract] OR IL-6[Title/Abstract] OR Anti-IL-6 Agent*[Title/Abstract])) OR ("Interleukin-6 Inhibitors"[Mesh])) AND (((((((((((((((( "heart failure"[MeSH Major Topic] NOT "decompensated heart failure"[All Fields]) NOT "acute heart failure"[All Fields]) NOT "Coronary Artery Disease"[MeSH Terms]) NOT "arrhythmias, cardiac"[MeSH Terms]) NOT "Cardio-Renal Syndrome"[MeSH Terms]) NOT "Heart Failure, Diastolic"[MeSH Terms]) NOT "HFpEF"[All Fields]) NOT "Heart Valve Diseases"[MeSH Terms]) NOT "Cardiomyopathies"[MeSH Terms]) NOT "edema, cardiac"[MeSH Terms]) NOT "dyspnea, paroxysmal"[MeSH Terms])) NOT "Atherosclerosis"[MeSH Terms]) NOT "Myocardial Ischemia"[MeSH Terms])) NOT animal[tiab] NOT cells[tiab] NOT (Abstract OR Case Reports OR Protocol OR Conference Abstracts OR Editorials OR Expert Opinion) AND (english[Filter]))

*EMBASE search string*

('heart failure'/mj NOT ('heart muscle ischemia'/de OR 'atherosclerosis'/de OR 'paroxysmal dyspnea'/de OR 'heart edema'/de OR 'cardiomyopathy'/de OR 'valvular heart disease'/de OR 'hfpef' OR 'diastolic heart failure'/de OR 'cardiorenal syndrome'/de OR 'heart arrhythmia'/de OR 'ischemic heart disease'/de OR 'acute heart failure' OR 'decompensated heart failure')) AND ('inhibitor* interleukin-6':ab,ti OR 'il-6 inhibitor*':ab,ti OR il6:ab,ti OR 'il 6':ab,ti OR 'anti-il-6 agent*':ab,ti OR 'interleukin 6 inhibitor'/exp) NOT (animal:ab,ti OR cells:ab,ti) NOT (abstract OR 'case reports' OR protocol OR 'conference abstracts' OR editorials OR 'expert opinion') AND [english]/lim

**Risk of bias assessment**

For studies evaluating IL-6 as a prognostic biomarker, a quality assessment of studies was conducted using the Quality in Prognosis Studies (QUIPS) tool (3) (4). Each study was evaluated across six domains: study participation, study attrition, prognostic factor measurement, confounding measurement and account, outcome measurement, analysis and reporting. Two reviewers (MC, CL) independently rated each domain as having low, moderate, or high risk of bias. Any discrepancies were resolved through discussion. For studies investigating the predictive or pharmacodynamic utility of IL-6, no validated or widely accepted tools for risk of bias assessment were identified in the literature. Consequently, a formal appraisal of bias was not feasible for these study types. This limitation was acknowledged and taken into account in the interpretation of findings.

**Supplementary tables**

Table S1 – Exclusion/inclusion criteria.

| **Category** | **Inclusion Criteria** | **Exclusion Criteria** |
| --- | --- | --- |
| **Baseline population** | - Patients with heart failure with reduced ejection fraction (HFrEF:LVEF < 50%) or equivalent. Including mixed HF cohorts if subgroup analysis of HFrEF is present.  - HF with systolic dysfunction.  - Age ≥ 6 years. | - Patients with preserved ejection fraction (HFpEF; LVEF ≥ 50%)    - Heart transplant (HTx).     - Congenital heart disease (e.g., tetralogy of Fallot, post-Fontan, systemic right ventricle).    - New onset of heart failure.  - Age < 6 years. |
| **Biomarkers** | - IL-6 used for pharmacodynamic (to assess treatment effect), prognostic (associated with outcomes regardless of therapy), and predictive purposes (to indicate the likelihood of response to a specific treatment). | - IL-6 used only for multivariable adjustments.    - IL-6 used for diagnostic purposes.     - No numerical data for IL-6. |
| **Outcome** | - All possible outcomes. | - New onset heart failure as an outcome in patients without pre-existing HF. |

Table S2 - Description of the three biomarker categories.

|  | **Time point** | **Treatment arm** | **Outcomes** | **Key use** | **Analyses** |
| --- | --- | --- | --- | --- | --- |
| **Prognostic** | Baseline;  Changes over time. | No | Yes  (disease progression or recurrence) | Used for risk stratification and guiding clinical decisions regarding monitoring and intervention. | Hazard Ratio (HR, from Cox Proportional Hazards Model);  Kaplan-Meier Survival analysis;  Delta value;  Initially at baseline, with potential follow-up assessments. |
| **Pharmacodynamic** | Multiple, including baseline. | Yes | IL-6 (changes) | Establishing proof-of-concept;  Assisting in dose selection;  Measuring response to medical products. | Measure at both baseline and follow-up;    T-test or ANOVA: comparing the difference of multiple timepoints. |
| **Predictive** | Baseline;  Changes over time | Yes | Treatment response | Selecting patients likely to respond to treatment or indicating potential adverse reactions. | Separate treatment analysis by group or with biomarker as an interaction with treatment effect. |

Table S3 -Pre-analytical and analytical characteristics of IL-6 measurement across included studies

.

| **Research Use Only** | | | | | | | | | |
| --- | --- | --- | --- | --- | --- | --- | --- | --- | --- |
| **Study** | **Assay** | **Category** | **Matrix** | **Anticoagulant** | **Processing delay** | **Centrifugation conditions** | **Storage temperature** | **Duration of storage** | **Freeze-thaw cycles** |
| Mommersteeg PMC. et al 2016 | ELISA (Diamed Eurogen) | ELISA | Serum | NA | NR | NR | -80°C | NR | 0 |
| Sunman H. et al 2018 | ELISA (Bender MedSystems) | ELISA | NR | NR | NR | NR | -80°C | NR | NR |
| Zeinab M. Helmy et al. 2013 | ELISA (not specified) | ELISA | NR | NR | NR | NR | NR | NR | NR |
| Jin L. et al 2026 | ELISA (not specified) | ELISA | Serum | NR | NR | 3000 r/min, radius 12 cm, for 15 min | NR | NR | NR |
| Zhang D et al. 2022 | ELISA (not specified) | ELISA | NR | NR | NR | NR | NR | NR | NR |
| Avgeropoulou C et al. 2005 | Instant ELISA Bender MedSystems | ELISA | Serum | NA | Within 1 month | NR | -80°C | NR | NR |
| Wei Li et al. 2021 | ELISA Biosource | ELISA | Plasma | NR | NR | NR | -80°C | NR | NR |
| Oyama Jet al. 2013 | ELISA Biosource | ELISA | Plasma | NR | NR | NR | NR | NR | 0 |
| Tasal A et al. 2014 | ELISA Biosource | ELISA | Serum | NA | NR | 1500 rpm for 5 min | -40°C | NR | NR |
| Kyrzopoulos S et al. 2005 | ELISA Diaclone | ELISA | Plasma | NR | NR | 3000 rpm, 15 min | -80°C | NR | 0 |
| Omidi S et al. 2020 | ELISA Diaclone | ELISA | NR | NR | NR | NR | NR | NR | NR |
| Przybyła A et al. 2011 | ELISA Diaclone | ELISA | Plasma | NR | NR | NR | NR | NR | NR |
| Yamada T et al. 2007 | ELISA Fijirevio | ELISA | NR | NR | NR | NR | NR | NR | NR |
| Orús J. et al 2000 | ELISA Medgenix | ELISA | Plasma | No anticoagulant (non-heparinized tube) | Within 2 h | NR | -20°C | NR | NR |
| Roig E. et al 1998 | ELISA Medgenix | ELISA | Plasma | NR | Within 2 h | NR | -20°C | NR | NR |
| Adamopoulos S et al. 2002 | ELISA R&D Systems | ELISA | Serum | NA | NR | 3000 rpm for 10 min | -70°C | NR | 0 |
| Boriani G et al. 2006 | ELISA R&D Systems | ELISA | Venous blood/plasma | NR | Within 1 h | Within 1 h | -80°C | NR | 0 |
| Byrkjeland R et al. 2011 | ELISA R&D Systems | ELISA | Serum | NA | 30 min. | 3000 g, 20 min | -80°C | NR | 0 |
| Caruso R et al. 2012 | ELISA R&D Systems | ELISA | Plasma | NR | NR | NR | NR | NR | NR |
| Deftereos S et al. 2014 | ELISA R&D Systems | ELISA | NR | NR | NR | NR | NR | NR | NR |
| Deswal A et al. 2001 | ELISA R&D Systems | ELISA | Plasma | EDTA | Within 30 min | NR | -70°C | NR | NR |
| Gielen S et al. 2003 | ELISA R&D Systems | ELISA | Serum | NA | NR | NR | -80°C | NR | 0 |
| Gwechenberger M. et al 2004 | ELISA R&D Systems | ELISA+ | Plasma | NR | NR | 4°C | -20°C | NR | NR |
| Kourea K et al. 2008 | ELISA R&D Systems | ELISA | Plasma | EDTA | Within 15 min. | 1000 x g, 4°C | -80°C | Until analysis | 1 |
| Lappegård KT et al. 2006 | ELISA R&D Systems | ELISA | Plasma | NR | NR | NR | -70°C | Until batch analysis | NR |
| Mayer B et al. 2005 | ELISA R&D Systems | ELISA | Plasma | NR | Immediately | 4°C | -20°C | Until analysis | NR |
| Niebauer J et al. 2005 | ELISA R&D Systems | ELISA | Plasma | EDTA | Immediately | 1300 rpm, 4°C | -80° | Until further analysis | NR |
| Parissis JT et al. 2005 | ELISA R&D Systems | ELISA | NR | NR | NR | NR | NR | NR | NR |
| Parissis JT et al. 2006 | ELISA R&D Systems | ELISA | Plasma | NR | NR | NR | NR | NR | NR |
| Parissis JT. et al 2009 (a) | ELISA R&D Systems | ELISA | Serum | NA | NR | NR | NR | NR | NR |
| Parissis JT. et al 2009 (b) | ELISA R&D Systems | ELISA | Plasma | NR | NR | NR | NR | NR | NR |
| Rubaj A et al. 2006 | ELISA R&D Systems | ELISA | Serum | NA | Immediately frozen after centriguation | 5000 rpm, 4°C, 10 min. | -80°C | Until assay | NR |
| Tousoulis D, et al. 2004 | ELISA R&D Systems | ELISA | Serum | NA | NR | NR | -20°C | Until assay | 0 |
| Zhao YT et al. 2009 | ELISA R&D Systems | ELISA | Plasma | EDTA | Within 15 min. | 1000 g, 4°C | -80°C | Until batch analysis | 0 |
| Tian H et al. 2021 | ELISA Shanghai Enzyme Union | ELISA | Serum | NA |  | 3000 rpm, 10 min. | NR | NR | NR |
| Duan HY et al. 2015 | ELISA Xinran Biotechnology | ELISA | Serum | NA | Within 30 min. | 3000 rpm, 15 min | NR | NR | NR |
| Marcucci R. et al 2006 | Biotrack IL6 HS (Amersham) | ELISA HS | Serum | NA | Immediately used for analysis | 1300 x g, 1o min. | NR | NR | NR |
| Bahrmann P et al. 2004 | Quantikine / Quantikine HS (R&D Systems) | ELISA HS | Plasma | NR | Within 30 min | 1000g, for 10 min | -80°C | NR | NR |
| Conraads VM et al. 2002 | Quantikine / Quantikine HS (R&D Systems) | ELISA HS | Plasma | EDTA | NR | NR | -20°C | NR | NR |
| Maeda K. et al 2000 | Quantikine / Quantikine HS (R&D Systems) | ELISA HS | Plasma | EDTA (1mg/mL) | Immediately placed on ice; centrifugation immediately implied | 4°C | -30°C | NR | NR |
| Moertl D et al. 2011 | Quantikine / Quantikine HS (R&D Systems) | ELISA HS | Plasma | EDTA | Immediately | 2000g, 4°C for 10 min | -70°C | Until analysis | NR |
| Node K et al. 2003 | Quantikine / Quantikine HS (R&D Systems) | ELISA HS | Plasma | EDTA | NR | NR | -80°C | NR | NR |
| Torre-Amione G. et al 1996 | Quantikine / Quantikine HS (R&D Systems) | ELISA HS | Plasma | NR | NR | NR | NR | NR | NR |
| Tsutamoto T. et al 1998 | Quantikine / Quantikine HS (R&D Systems) | ELISA HS | Plasma | EDTA, 1 mg/mL | NR | 3000 rpm,15 min, 4°C | -30°C | Until assayed | 0 |
| Tsutamoto T et al. 2000 | Quantikine / Quantikine HS (R&D Systems) | ELISA HS | Plasma | EDTA, 1 mg/mL | NR | 3000 rpm,15 min, 4°C | -30°C | Until assayed | 0 |
| Tsutamoto T. et al 2007 | Quantikine / Quantikine HS (R&D Systems) | ELISA HS | Plasma | EDTA, 1 mg/mL + aprotinin 500 KIU/mL | NR | 3000 rpm,15 min, 4°C | -30°C | NR (until assay) | NR |
| Eleuteri E et al. 2013 | R&D Systems HS kit | ELISA HS | Serum | NA | Immediately | NR | -80°C | Until analysis | NR |
| Gombos T. et al 2012 | R&D Systems HS kit | ELISA HS | Serum | NA | NR | NR | -70°C | NR | NR |
| Seifert M et al. 2007 | R&D Systems HS kit | ELISA HS | Plasma | NR | NR | 3000 rpm | -70°C | NR | NR |
| Theodorakis GN et al. 2006 | R&D Systems HS kit | ELISA HS | Serum | NA | Within 30 min | NR | -80°C | NR | NR |
| Pullen PR et al. 2008 | Plate reader 420 nm | Enzyme immunoassay (EIA), Cayman Chemical | Serum | NA | NR | NR | -80°C | NR | NR |
| Pullen PR et al. 2010 | Plate reader 420 nm | Enzyme assay | Serum | NA | NR | NR | -80°C | NR | NR |
| Sola S et al. 2005 | Plate reader 420 nm | Enzyme assay | Serum | NA | NR | NR | -80°C | NR | NR |
| Sola S et al. 2006 | Plate reader 420 nm | Enzyme assay | Serum | NA | NR | NR | -80°C | NR | NR |
| Nessler J et al. 2008 | Immunoenzymatic Milenia | Immunoenzyme | Serum | NA | NR | NR | NR | NR | NR |
| Nodari S et al. 2009 | Immunoenzymatic spectrophotometric | Immunoenzyme | Serum | NA | Within 1h | 1700g, 4°C for 15 min | -80°C | NR | NR |
| Nodari S et al. 2011 | Immunoenzymatic spectrophotometric | Immunoenzyme | Serum | NA | Within 1h | 1700g, 4°C for 15 min | -80°C | NR | NR |
| Ogino K et al. 2010 | ELEIA Rumipulse-f (Fujirebio) | Immunoenzyme | Serum | NA | NR | NR | NR | NR | NR |
| Gotzmann M et al. 2023 | LegendPlex (BioLegend) | Multiplex | NR | NR | NR | NR | NR | NR | NR |
| Markousis-Mavrogenis G. et al 2019 | Luminex platform | Multiplex | Plasma | NR | NR | NR | NR | NR | NR |
| Fernandes-Silva MM et al. 2017 | Milliplex MAP (Luminex) | Multiplex | Serum | NA | Immediately | 2000g, 4°C for 10 min | -70°C | Until analysis | NR |
| Askevold ET. et al 2013 | Randox Multiplex | Multiplex | Plasma | NR | NR | NR | NR | NR | NR |
| Ge T et al. 2023 | Radioimmunoassay Wuhan Saipei | RIA | Serum | NA | NR | NR | NR | NR | NR |
| **In Vitro Diagnostic** | | | | | | | | | |
| **Study** | **Assay** | **Category** | **Matrix** | **Anticoagulant** | **Processing delay** | **Centrifugation conditions** | **Storage temperature** | **Duration of storage** | **Freeze-thaw cycles** |
| Feiereisen P et al. 2013 | Immulite 2000 (Siemens) | Automated immunoassay | Serum | NA | NR | NR | NR | NR | NR |
| Boffa GM. et al 2009 | IMMULITE One (Medical System) | Automated immunoassay | Serum | NA | NR | NR | NR | NR | NR |
| Rauchhaus M. et al 2000 | Immulite Random Access Analyzer | Automated immunoassay | Plasma | EDTA | NR | NR | NR | NR | NR |
| Docherty KF. et al 2025 | Cobas e411 (Roche) | Chemiluminescence | Serum | NA | NR | NR | NR | NR | NR |
| Defilippi CR. et al 2022 | Siemens Diagnostic | Immunoassay | Serum | NA | NR | NR | -80°C | NR | 2 |
| Wood N. et al 2026 | MSD V-PLEX Human Cytokine 30-Plex | Immunoassay | Serum | NA | NR | NR | NR | NR | NR |
| **Other** | | | | | | | | | |
| **Study** | **Assay** | **Category** | **Matrix** | **Anticoagulant** | **Processing delay** | **Centrifugation conditions** | **Storage temperature** | **Duration of storage** | **Freeze-thaw cycles** |
| Boxer RS. et al 2011 | NR | NR | Serum | NA | NR | NR | -70°C | NR | NR |
| Krum H. et al 2007 | NR | NR | NR | NR | NR | NR | NR | NR | NR |
| Stieger P. et al 2018 | NR | NR | Serum | NA | NR | NR | NR | NR | NR |
| Tian X. et al 2024 | NR | NR | Serum | NA | NR | NR | NR | NR | NR |
| Xie L. et al 2024 | NR | NR | NR | NR | NR | NR | NR | NR | NR |
| de Meirelles LR et al. 2014 | NR | NR | NR | NR | NR | NR | NR | NR | NR |
| Bielecka-Dabrowa et al 2009 | NR | NR | Serum | NA | NR | NR | -70°C | NR | NR |
| Bielecka-Dabrowa et al 2013 | Sandwich ELISA | Beckman Coulter | NR | NR | NR | NR | NR | NR | NR |
| Kobayashi N et al. 2003 | NR | NR | NR | NR | NR | NR | NR | NR | NR |
| Wang D et al. 2020 | Immunoturbidimetry | NR | NR | NR | NR | NR | NR | NR | NR |
| Bielecka-Dabrowa et al 2013 | Sandwich ELISA | Beckman Coulter | Plasma | EDTA | NR | 15 min | -70°C | NR | NR |

Freeze-thaw cycles = 0: samples were stored frozen and thawed only once, immediately before analysis.

Table S4 - Summary of literature on IL-6 as prognostic biomarker in patients with heart failure with reduced ejection fraction (HFrEF)

| **All-cause death** (n=14)† | | | | | | | | |
| --- | --- | --- | --- | --- | --- | --- | --- | --- |
|  | Univariate (n=11) | | Multivariate (n=14) | | Adjustment with Natriuretic peptides (n=7) | | Adjustment with inflammatory markers (n=8) | |
| Prognostic value | **Yes** | **No** | **Yes** | **No** | **Yes** | **No** | **Yes** | **No** |
| N studies | 9 | 2 | 7 | 7 | 3 | 4 | 3 | 5 |
| N participants | 7644 | 147 | 6196 | 1976 | 3165 | 1696 | 613 | 1829 |
| **CV death** (n = 4)* | | | | | | | | |
|  | Univariate (n=4) | | Multivariate (n=4) | | Adjustment with Natriuretic peptides (n=3) | | Adjustment with inflammatory markers (n=2) | |
| Prognostic value | **Yes** | **No** | **Yes** | **No** | **Yes** | **No** | **Yes** | **No** |
| N studies | 4 | 0 | 3 | 1 | 3 | 1 | 1 | 1 |
| N participants | 11771 | 0 | 10319 | 1452 | 9442 | 1452 | 5050 | 1452 |
| **HF composite** (n=13)‡ | | | | | | | | |
|  | Univariate (n=12) | | Multivariate (n=13) | | Adjustment with Natriuretic peptides (n=10) | | Adjustment with inflammatory markers (n=6) | |
| Prognostic value | **Yes** | **No** | **Yes** | **No** | **Yes** | **No** | **Yes** | **No** |
| N studies | 9 | 3 | 6 | 7 | 4 | 6 | 2 | 4 |
| N participants | 12474 | 498 | 10546 | 2466 | 8130 | 2252 | 5150 | 1991 |
| **Hospitalizations** (n = 4)¥ | | | | | | | | |
|  | Univariate (n=4) | | Multivariate (n=4) | | Adjustment with Natriuretic peptides (n=2) | | Adjustment with inflammatory markers (n=2) | |
| Prognostic value | **Yes** | **No** | **Yes** | **No** | **Yes** | **No** | **Yes** | **No** |
| N studies | 2 | 2 | 0 | 4 | 0 | 2 | 0 | 2 |
| N participants | 6502 | 2397 | 0 | 8899 | 0 | 6502 | 0 | 6502 |

†: Torre-Amione G. et al 1996, Tsutamoto T. et al 1998, Maeda K. et al  2000, Rauchhaus M. et al 2000, Deswal A. et al 2001, Marcucci R. et al 2006, Tsutamoto T. et al 2007, Boffa GM. et al 2009, Boxer RS. et al  2011, Askevold ET. et al 2013, Bielecka-Dabrowa A. et al  2013, Mommersteeg PMC. et al 2016, Markousis-Mavrogenis G. et al 2019, Docherty KF. et al 2025
* : Askevold ET. et al 2013 , Markousis-Mavrogenis G. et al  2019  , Defilippi CR. et al 2022, Docherty KF. et al 2025
‡: Roig E. et al 1998, Maeda K. et al  2000,  Orús J. et al 2000, Gwechenberger M. et al  2004 , Marcucci R. et al  2006, Boffa GM. et al  2009  Parissis JT. et al 2009 (a) Parissis JT. et al 2009 (b), Gombos T. et al  2012, Askevold ET. et al 2013, Markousis-Mavrogenis G. et al  2019, Defilippi CR. et al 2022, Docherty KF. et al 2025
¥: Askevold ET. et al 2013, Bielecka-Dabrowa A. et al  2013  , Markousis-Mavrogenis G. et al  2019  , Defilippi CR. et al 2022

Table S5 - Risk of bias assessment of included studies using the QUIPS tool

| **Author, year** | **QUIPS domains** | | | | | |
| --- | --- | --- | --- | --- | --- | --- |
|  | **1. Study Participation** | **2. Study Attrition** | **3. Prognostic Factor Measurement** | **4. Outcome Measurement** | **5. Study Confounding** | **6. Statistical Analysis and Reporting** |
| Askevold ET. et al 2013 | moderate | high | low | moderate | moderate | low |
| Bielecka-Dabrowa A. et al 2013 | moderate | moderate | low | low | moderate | moderate |
| Boffa GM. et al 2009 | low | low | low | high | moderate | low |
| Boxer RS. et al 2011 | low | high | low | low | low | moderate |
| Defilippi CR. et al 2022 | low | low | moderate | low | moderate | low |
| Deswal A. et al 2001 | low | low | moderate | low | moderate | low |
| Docherty K. et al 2025 | moderate | high | moderate | low | low | low |
| Gombos T. et al 2012 | moderate | low | low | moderate | low | low |
| Gwechenberger M. et al 2004 | moderate | low | low | moderate | low | moderate |
| Maeda K. et al 2000 | moderate | moderate | low | moderate | low | low |
| Marcucci R. et al 2006 | moderate | low | low | high | moderate | low |
| Markousis-Mavrogenis G. et al 2019 | moderate | moderate | low | moderate | low | low |
| Mommersteeg PMC. et al 2016 | low | high | low | low | low | low |
| Orús J. et al 2000 | moderate | high | high | high | moderate | moderate |
| Parissis JT. et al 2009 (a) | moderate | high | moderate | moderate | moderate | high |
| Parissis JT. et al 2009 (b) | moderate | low | low | moderate | low | low |
| Rauchhaus M. et al 2000 | high | moderate | moderate | moderate | moderate | low |
| Roig E. et al 1998 | high | high | high | high | moderate | high |
| Tsutamoto T. et al 1998 | high | high | high | moderate | high | moderate |
| Torre-Amione G. et al 1996 | moderate | moderate | moderate | high | moderate | moderate |
| Tsutamoto T. et al 2007 | moderate | low | low | moderate | low | low |

Table S6 – Comparison between treatment-induced IL-6 changes and available clinical outcome evidence.

| **Treatment** | **Change in IL-6** | **Cardiovascular outcomes in HFrEF** |
| --- | --- | --- |
| Rosuvastatin | No change^1^ | No improvement^a,b^ |
| Dapagliflozin | No significant change ^2^ | Improvement^c^ |
| Carvedilol | Decrease^3^ | Improvement^d^ |
| Metoprolol | Decrease^4,5^ | Improvement^e^ |
| Losartan | Decrease^6^ | Improvement^f^ |
| Candesartan cilexetil | Decrease^7^ | Improvement^g^ |
| Levosimendan | Decrease^8-12^ | Inconsistent results^h,i^ |
| Darbepoetin alfa | Decrease^13^ | No improvement^l^ |
| Colchicine | Decrease^14^ | No improvement (pilot study)^m^ |
| Pentoxifylline | No change^15^ | NA (only few very small pilot studies)^n,o^ |
| Cardiac resynchronization therapy | No consistent changes^16-20^ | Improvement^p,q,r^ |
| Polyunsaturated fatty acids | No constistent changes^21-24^ | Improvement^s^ |

^1^Krum H et al., 2007; ^2^ Docherty KF et al., 2025; ^3^Nessler J et al., 2008; ^4^Tian H et al., 2021; ^5^Mayer B et al., 2005; ^6^Ogino K et al., 2010; ^7^Tsutamoto T et al., 2000; ^8^Avgeropoulou C et al., 2005; ^9^Parissi JT 2005 et al., 2005; ^10^Parissis JT et al. 2006; ^11^Tasal A et al., 2014; ^12^KyrzopoulosS et al., 2005; ^13^Kourea K et al., 2008; ^14^Deftereos S et al., 2014; ^15^Bahrmann P et al., 2004; ^16^Lappegard KT et al., 2006; ^17^Seifert M et al., 2007; ^18^Boriani G et al., 2006; ^19^Theodorakis GN et al., 2006; ^20^Przybyla A et al., 2011; ^21^Nodari Set al., 2009; ^22^Zhao et al., 2009; ^23^Nodari S et al., 2011; ^24^Moertl et al., 2011.

^a^Kjekshus J, Apetrei E, Barrios V, et al. Rosuvastatin in older patients with systolic heart failure. *N Engl J Med*. 2007;357(22):2248-2261. doi:10.1056/NEJMoa0706201
^b^Tavazzi L, Maggioni AP, Marchioli R, et al. Effect of rosuvastatin in patients with chronic heart failure (the GISSI-HF trial): a randomised, double-blind, placebo-controlled trial. *Lancet*. 2008;372(9645):1231-1239. doi:10.1016/S0140-6736(08)61240-4
^c^Docherty KF, McDowell K, Welsh P, et al. Interleukin-6 in Heart Failure With Reduced Ejection Fraction and the Effect of Dapagliflozin: An Exploratory Analysis of the Dapagliflozin and Prevention of Adverse Outcomes in Heart Failure Trial. *JACC Heart Fail*. 2025;13(7):102393. doi:10.1016/j.jchf.2024.12.012
^d^Packer M, Coats AJ, Fowler MB, et al. Effect of carvedilol on survival in severe chronic heart failure. *N Engl J Med*. 2001;344(22):1651-1658. doi:10.1056/NEJM200105313442201
^e^Effect of metoprolol CR/XL in chronic heart failure: Metoprolol CR/XL Randomised Intervention Trial in Congestive Heart Failure (MERIT-HF). *Lancet*. 1999;353(9169):2001-2007.
^f^Konstam MA, Neaton JD, Dickstein K, et al. Effects of high-dose versus low-dose losartan on clinical outcomes in patients with heart failure (HEAAL study): a randomised, double-blind trial. *Lancet*. 2009;374(9704):1840-1848. doi:10.1016/S0140-6736(09)61913-9
^g^Pfeffer MA, Swedberg K, Granger CB, et al. Effects of candesartan on mortality and morbidity in patients with chronic heart failure: the CHARM-Overall programme. *Lancet*. 2003;362(9386):759-766. doi:10.1016/s0140-6736(03)14282-1
^h^Packer M, Colucci W, Fisher L, et al. Effect of levosimendan on the short-term clinical course of patients with acutely decompensated heart failure. *JACC Heart Fail*. 2013;1(2):103-111. doi:10.1016/j.jchf.2012.12.004
^i^García-González MJ, Aldea Perona A, Lara Padron A, et al. Efficacy and safety of intermittent repeated levosimendan infusions in advanced heart failure patients: the LAICA study. *ESC Heart Fail*. 2021;8(6):4820-4831. doi:10.1002/ehf2.13670
^l^Swedberg K, Young JB, Anand IS, et al. Treatment of anemia with darbepoetin alfa in systolic heart failure. *N Engl J Med*. 2013;368(13):1210-1219. doi:10.1056/NEJMoa1214865
^m^Pascual-Figal D, Núñez J, Pérez-Martínez MT, et al. Colchicine in acutely decompensated heart failure: the COLICA trial. *Eur Heart J*. 2024;45(45):4826-4836. doi:10.1093/eurheartj/ehae538
^n^Skudicky D, Bergemann A, Sliwa K, Candy G, Sareli P. Beneficial effects of pentoxifylline in patients with idiopathic dilated cardiomyopathy treated with angiotensin-converting enzyme inhibitors and carvedilol: results of a randomized study. *Circulation*. 2001;103(8):1083-1088. doi:10.1161/01.cir.103.8.1083
^o^Sliwa K, Woodiwiss A, Kone VN, et al. Therapy of ischemic cardiomyopathy with the immunomodulating agent pentoxifylline: results of a randomized study. *Circulation*. 2004;109(6):750-755. doi:10.1161/01.CIR.0000112568.48837.60
^p^Cleland JG, Daubert JC, Erdmann E, et al. The effect of cardiac resynchronization on morbidity and mortality in heart failure. *N Engl J Med*. 2005;352(15):1539-1549. doi:10.1056/NEJMoa050496
 ^q^Bristow MR, Saxon LA, Boehmer J, et al. Cardiac-resynchronization therapy with or without an implantable defibrillator in advanced chronic heart failure. *N Engl J Med*. 2004;350(21):2140-2150. doi:10.1056/NEJMoa032423
^r^Tang AS, Wells GA, Talajic M, et al. Cardiac-resynchronization therapy for mild-to-moderate heart failure. *N Engl J Med*. 2010;363(25):2385-2395. doi:10.1056/NEJMoa1009540
^s^Tavazzi L, Maggioni AP, Marchioli R, et al. Effect of n-3 polyunsaturated fatty acids in patients with chronic heart failure (the GISSI-HF trial): a randomised, double-blind, placebo-controlled trial. *Lancet*. 2008;372(9645):1223-1230. doi:10.1016/S0140-6736(08)61239-8

Table S7 - Distribution of LVEF% cutoffs in the 80 selected publications.

| **LVEF cutoff (%)** | **N studies** | **N studies cumulative** |
| --- | --- | --- |
| <25 | 1 | 1 |
| <30 | 4 | 5 |
| <=30 | 3 | 8 |
| <35 | 6 | 14 |
| <=35 | 5 | 19 |
| <40 | 26 | 45 |
| <=40 | 15 | 60 |
| <45 | 7 | 67 |
| <=45 | 6 | 73 |
| <50 | 4 | 77 |
| <=50 | 1 | 78 |
| Advanced decompensated HF, LVEF% not reported | 2 | 80 |
| **Tot** | **80** | **80** |

**Supplementary figures**

Figure S1: Summary of Risk of Bias 

 

**References of Supplementary Appendix**

1.	Huang X, Lin J, Demner-Fushman D. Evaluation of PICO as a knowledge representation for clinical questions. AMIA Annu Symp Proc. 2006;2006:359-63.

2.	Ye, Y; Colombo, M; Meessen, J; Hajizadeh, N; De Vore, L; Sigarchian, H. G; Peters, C; Krovvidi, S; Meresh, S; Hasson, L; Jakka, S. R; Tyl, B; on behalf of the iCARE4CVD consortium. Automated Full-text screening and accelerated reviews using large language models with Context-Aware Agents: An exploratory analysis in biomarker research. Manuscript submitted for publication to European Heart Journal - Digital Health. 2025.

3.	Riley RD, Hayden JA, Steyerberg EW, Moons KG, Abrams K, Kyzas PA, et al. Prognosis Research Strategy (PROGRESS) 2: prognostic factor research. PLoS Med. 2013;10(2):e1001380.

4.	Hayden JA, van der Windt DA, Cartwright JL, Côté P, Bombardier C. Assessing bias in studies of prognostic factors. Ann Intern Med. 2013;158(4):280-6.

**List of reviewers and investigators not appearing as coauthors**

| **Institution** | **Reviewers and investigators** |
| --- | --- |
| **AI-tool development** | |
| Capsico Health | Laurent Hasson, SaiRamesh Jakka, Shloke Meresh, Supraja Krovvidi, Finn Vos |
| Thomas More | Hajar Ghaem Sigarchian |
| Evotec | Lauren De Vore |
| **Binning & Information extraction** | |
| University of Maastricht | Arantxa Barandiarán Aizpurua, Christof Peters |
| Universitätsklinikum Aachen | Dirk Müller-Wieland |
| Istituto di Ricerche Farmacologiche Mario Negri IRCCS | Ece Buldurluoglu, Silvia Maria Balosso |
| Novo Nordisk | Katarina Pardali |
| Evotec | Sadhana Panzade, Sara Klas |
| Medizinische Universität Wien | Noemi Pavo |
| Institute National De La Santé Et De La Recherche Médicale | Paul Balfanz |
| **Risk of bias assessment** | |
| Istituto di Ricerche Farmacologiche Mario Negri IRCCS | Michela Cinquini, Carolina Lavazza |
| **Search string in Medline and Embase** | |
| Istituto di Ricerche Farmacologiche Mario Negri IRCCS | Veronica Andrea Fittipaldo, Maria Chiara Bassi |
| **Reviewers** | |
| German Foundation for the Chronically Ill (FCI) / Deutsche Stiftung für chronisch Kranke | Jana Seuthe |
